# The Effect of Walking on Depressive and Anxiety Symptoms: Systematic Review and Meta-Analysis

**DOI:** 10.2196/48355

**Published:** 2024-07-23

**Authors:** Zijun Xu, Xiaoxiang Zheng, Hanyue Ding, Dexing Zhang, Peter Man-Hin Cheung, Zuyao Yang, King Wa Tam, Weiju Zhou, Dicken Cheong-Chun Chan, Wenyue Wang, Samuel Yeung-Shan Wong

**Affiliations:** 1 Jockey Club School of Public Health and Primary Care, The Chinese University of Hong Kong, Hong Kong, China (Hong Kong)

**Keywords:** walking, depression, anxiety, systematic review, meta-analysis

## Abstract

**Background:**

Previous literature lacks summative information on the mental health benefits achieved from different forms of walking.

**Objective:**

The aim of this study was to assess the effectiveness of different forms of walking in reducing symptoms of depression and anxiety.

**Methods:**

This was a systematic review and meta-analysis of randomized controlled trials (RCTs) assessing the effects of walking on depressive and anxiety symptoms. MEDLINE, Cochrane Central Register of Controlled Trials (CENTRAL), Embase, PsycINFO, Allied and Complementary Medicine Database (AMED), CINAHL, and Web of Science were searched on April 5, 2022. Two authors independently screened the studies and extracted the data. Random-effects meta-analysis was used to synthesize the data. Results were summarized as standardized mean differences (SMDs) with 95% CIs in forest plots. The risk of bias was assessed by using the Cochrane Risk of Bias tool.

**Results:**

This review included 75 RCTs with 8636 participants; 68 studies reported depressive symptoms, 39 reported anxiety symptoms, and 32 reported both as the outcomes. One study reported the results for adolescents and was not included in the meta-analysis. The pooled results for adults indicated that walking could significantly reduce depressive symptoms (RCTs: n=44; SMD −0.591, 95% CI −0.778 to −0.403; *I*^2^=84.8%; τ^2^=0.3008; *P*<.001) and anxiety symptoms (RCTs: n=26; SMD −0.446, 95% CI −0.628 to −0.265; *I*^2^=81.1%; τ^2^=0.1530; *P*<.001) when compared with the inactive controls. Walking could significantly reduce depressive or anxiety symptoms in most subgroups, including different walking frequency, duration, location (indoor or outdoor), and format (group or individual) subgroups (all *P* values were <.05). Adult participants who were depressed (RCTs: n=5; SMD −1.863, 95% CI −2.764 to −0.962; *I*^2^=86.4%; τ^2^=0.8929) and those who were not depressed (RCTs: n=39; SMD −0.442, 95% CI −0.604 to −0.280; *I*^2^=77.5%; τ^2^=0.1742) could benefit from walking effects on their depressive symptoms, and participants who were depressed could benefit more (*P*=.002). In addition, there was no significant difference between walking and active controls in reducing depressive symptoms (RCTs: n=17; SMD −0.126, 95% CI −0.343 to 0.092; *I*^2^=58%; τ^2^=0.1058; *P*=.26) and anxiety symptoms (14 RCTs, SMD −0.053, 95% CI −0.311 to 0.206, *I*^2^=67.7%, τ^2^=0.1421; *P*=.69).

**Conclusions:**

Various forms of walking can be effective in reducing symptoms of depression and anxiety, and the effects of walking are comparable to active controls. Walking can be adopted as an evidence-based intervention for reducing depression and anxiety. More evidence on the effect of low-intensity walking is needed in the future.

## Introduction

Depression and anxiety are common mental disorders, with the global prevalence of depression in 2023 being 5% and that of anxiety disorders being 4% [1-3]. About 18.4% and 33.7% of people experience depression and anxiety disorders, respectively, at some time in their lives [4-6]. Globally, the age-standardized, disability-adjusted life-year rates for depressive disorders and anxiety disorders were 585 and 359 per 100,000 population, respectively, in 2019, which accounted for the two largest proportions of mental disorder disability-adjusted life-years [7]. In addition, there are many more people experiencing subthreshold depression and anxiety [8,9], especially during the COVID-19 pandemic. Interventions are available to treat depression and anxiety or reduce their symptoms, including antidepressants, psychotherapy, and physical exercise [10]. Walking, as one type of physical exercise, is considered safe, convenient, and noninvasive; it has low or no costs and does not require any special equipment or training, unlike some other forms of exercise [11]. It is a good intervention candidate that can be easily implemented, particularly for people with subthreshold depression and anxiety or patients who may not have access to or prefer not to take medication or therapy.

In recent years, more and more studies on walking have been conducted and have attracted attention from the public [12-14]. Prior systematic reviews found walking to have benefits for various aspects of health, such as improvements in cardiorespiratory health [15], blood pressure [16], cholesterol levels [17], chronic pain [18], and weight loss [19], as well as reductions in the risk for diabetes [20] and all-cause mortality [21].

Apart from its benefits for physical health, previous systematic reviews and meta-analyses were conducted to measure the effect of walking on depressive symptoms. A previous meta-analysis of 8 randomized controlled trials (RCTs) with a total of 341 participants who were depressed found that walking interventions had a significant, large, standardized mean difference (SMD) of −0.86 for depressive symptoms in adult participants [22]. In 2015, another meta-analysis with a total of 110 adult participants indicated a significant benefit of outdoor group walking for depressive symptoms, with an SMD of −0.67 [23]. Other meta-analyses only focused on a specific group of people or one form of walking, such as people with postpartum depression and nature walking [24,25]. These previous studies only included a small number of RCTs with a small total number of participants or focused on a specific population or form of walking. More RCTs on walking have been published in recent years but have not been included in any systematic reviews; a review of these RCTs, which provide more robust evidence, would allow for a more comprehensive understanding of the effects of walking. In addition, previous reviews did not distinguish between different types of control groups. More importantly, previous meta-analyses lacked detailed information on other forms of walking that can provide mental health benefits, such as indoor and outdoor walking, individual and group walking, walking facilitated by equipment (eg, pedometers), and nonfacilitated walking. Furthermore, it remains unclear whether specific factors of walking, such as various walking characteristics (eg, duration, intensity, frequency, and pace) or walking with or without instructions, affect the results of walking interventions. Understanding the information on different forms of walking can help with informing health care professionals about how to promote walking in a very specific way, supporting mental health services in community and clinical settings, and informing public health policies on walking promotion [26]. This systematic review and meta-analysis was undertaken to assess the effects of walking and its different forms on reducing symptoms of depression and anxiety via comparisons with inactive controls and active controls, using the latest available evidence.

## Methods

This review was registered with PROSPERO (International Prospective Register of Systematic Reviews; CRD42021247983) and was conducted in accordance with the PRISMA (Preferred Reporting Items for Systematic Reviews and Meta-Analyses) statement [27].

### Literature Search

Systematic searches were restricted to English-language articles in electronic databases that were published from the date that the database was established to April 5, 2022. The seven databases searched included MEDLINE (PubMed), Cochrane Central Register of Controlled Trials (CENTRAL), Embase, PsycINFO, Allied and Complementary Medicine Database (AMED), CINAHL, and Web of Science. The strategies used for the search included the following: (walk* OR pedometer* OR step count) AND (depress* OR anxiety OR anxio*) AND (randomized controlled trial OR controlled clinical trial). The full search strategies can be found in Table S1 in Multimedia Appendix 1. Articles from relevant systematic reviews and meta-analyses were included. Backward and forward reference searching were also performed on the articles identified from relevant systematic reviews and meta-analyses.

Four authors (ZX, XZ, HD, and PMHC) were involved in the article screening, using Covidence (Veritas Health Innovation). Two authors independently performed the screening of titles and abstracts after Covidence automatically removed the duplicate articles. Two authors then reviewed the full texts of short-listed articles. With regard to articles for which full texts were not accessible on the internet, we sent emails to these articles’ authors to request full texts directly. Another author (DZ) was involved in the discussions to resolve any discrepancies in the title and abstract screening and the full-text review.

### Eligibility Criteria

The detailed inclusion and exclusion criteria are summarized in Table 1.

**Table 1.** Inclusion and exclusion criteria of this systematic review, which assessed the effect of walking on depression and anxiety.

Category	Inclusion criteria	Exclusion criteria
Population	No restriction on the population	None
Intervention	Any kind of walking intervention	A mixed intervention, such as combining walking with other physical activities, supplements, or psychosocial intervention, and 1-bout or 1-day walking
Control	No intervention or interventions other than walking	Control group containing walking
Outcome	Any scale measuring symptoms of depression and anxiety at baseline and after intervention	Scales measuring overall psychological well-being instead of depressive and anxiety symptoms
Study design	Randomized controlled trial	Interventional study without a control group, case report, cross-sectional study, cohort study, case-control study, qualitative study, and review
Article type	Research paper	Conference paper or letter
Language	English	Not English
Other	Full text available	Full text not available

### Data Extraction

Three authors (ZX, XZ, and HD) were involved in the data extraction. Two authors independently extracted data from all eligible articles. The extracted data included the title, first author, year of publication, country, participant characteristics (age, gender, and health condition), details of the intervention (eg, intervention duration and the intensity, frequency, and duration of each walk), and outcomes (symptoms of depression and anxiety measured by scales).

The Cochrane Collaboration Recommendations assessment tool was used to evaluate the risk of bias for the included studies [28]. Due to the nature of the walking interventions, the participants could not be blinded to the group allocation. Therefore, the item “blinding of participants and personnel” was not included in this study. The level of the risk of bias for each item and the overall risk of bias were classified as low, unclear, and high. If the risk of bias level for all items was classified as low, the overall risk of bias for the study was defined as low. If the risk of bias level for at least one item was rated as high, the overall risk of bias was defined as high. If the risk of bias level for at least one item was unclear and no item was rated as high risk, the overall risk of bias was defined as unclear [29]. For any discrepancies in data extraction and the assessed risk of bias between the aforementioned authors, another author (DZ) was referred to and made a decision after discussion.

### Statistical Analysis

The effect size SMDs for depressive and anxiety symptoms were initially calculated from the mean differences and SDs between the baseline and postintervention data of each study. If the SD of the mean difference was not available in a study, it was calculated from SEs and 95% CIs, in accordance with the *Cochrane Handbook for Systematic Reviews of Interventions* [28]. When the SD could not be calculated, the median correlation coefficients between baseline and postintervention scores, which were calculated by using the median correlations of studies without missing data, were used to estimate the missing SDs for scales of depression and anxiety [28].

The nonwalking comparators were categorized as inactive controls, active controls, and other controls. Inactive controls included no intervention, waitlists, usual care that did not involve treating depression or anxiety, brief instructions on physical activity, and placebo meetings, in accordance with a previous meta-analysis and a decision framework about control conditions for RCTs in psychiatry [30,31]. Active controls were defined as evidence-based interventions that have been shown to have positive effects on depression and anxiety, such as moderate-intensity exercise (eg, strengthening exercise, resistance exercise, swimming, cycling, jogging, Pilates, and stabilization exercise), yoga [32], tai chi [33], meditation [34], cognitive behavioral therapy [35], stress management training [36], art therapy [37], and social interaction [38]. Other controls included light exercise (eg, low-intensity stretching or relaxation exercises), nutrition-related control, and regular education, which lack enough evidence of their effects on depression and anxiety. The primary analysis was the comparison between walking and inactive controls, and the secondary analysis compared walking with active controls and other controls.

The meta-analysis was performed by using the random-effects model [39]. The SMD was used as the outcome to pool the results of the different scales. The SMDs of each study, their 95% CIs, and the pooled results were also presented in forest plots. *I*² and τ² were reported to indicate the heterogeneity of studies [40]. Small-study effects, which mainly include publication bias, were assessed by using funnel plots and the Egger test [41,42]. A subgroup analysis was conducted according to the different forms or characteristics of the walking interventions. The definition of each subgroup is summarized in Table S2 in Multimedia Appendix 1. The forms of walking with a *P* value of <.05 in the subgroup analysis were entered into the multivariate meta-regression. The significance level of the between-group differences was computed, and a 2-tailed *P* value of <.05 was considered statistically significant. All statistical analyses were performed by using Stata (version 16.0; StataCorp LLC).

### Ethical Considerations

This was a systematic review and meta-analysis and thus did not require ethical approval. This work was conducted following the World Medical Association's Declaration of Helsinki.

## Results

### Study Selection

**Figure 1.**
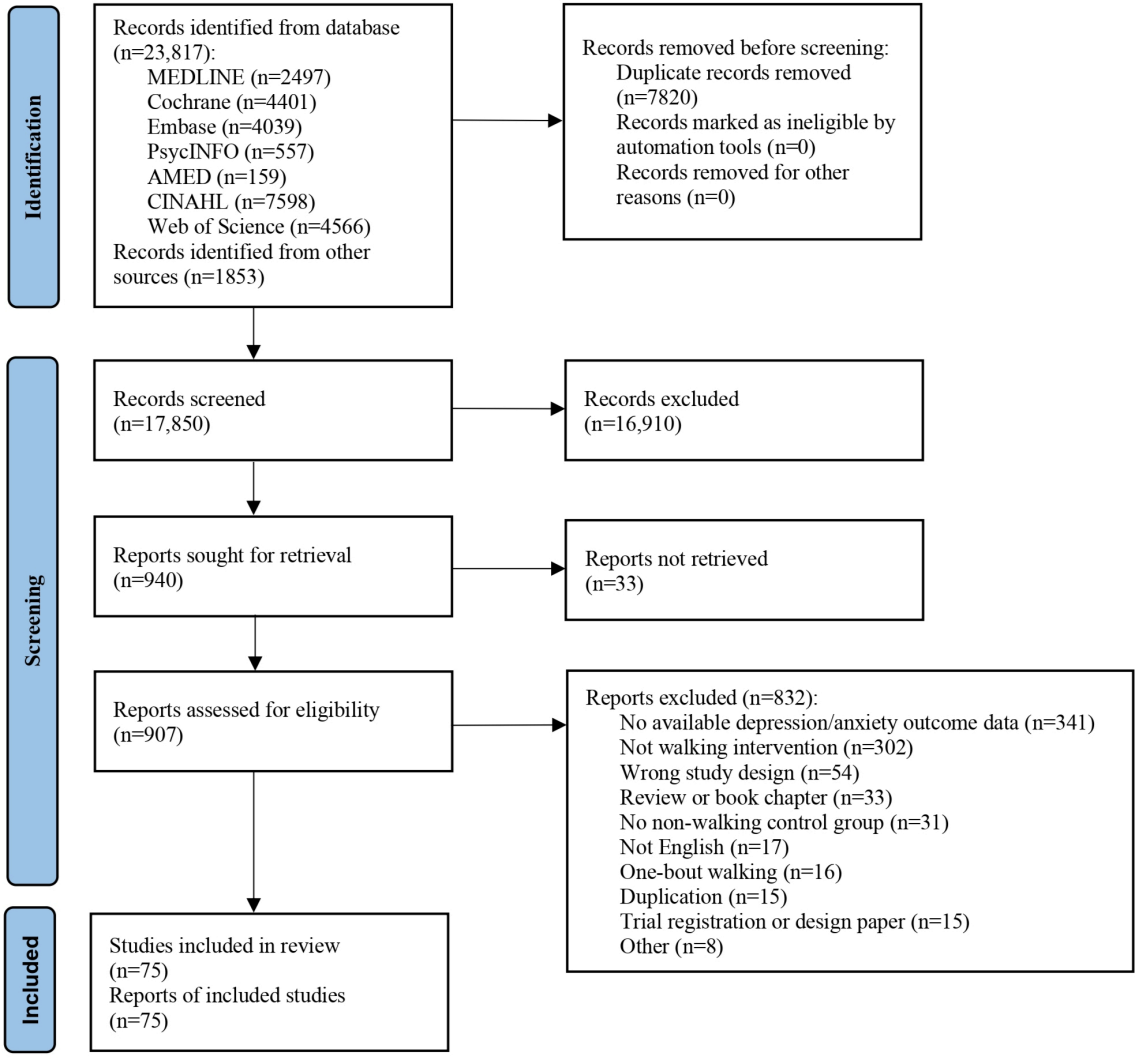
PRISMA (Preferred Reporting Items for Systematic Reviews and Meta-Analyses) flow diagram of this systematic review, which assessed the effects of walking and its different forms on reducing symptoms of depression and anxiety. The flow diagram depicts the identification of studies via databases and other sources. AMED: Allied and Complementary Medicine Database.

Figure 1 shows the flow diagram of the study selection process. A total of 23,817 records were identified from the 7 databases, and 1853 records were identified from relevant systematic reviews and via backward and forward reference searching. After removing 7820 duplicate records, 17,850 records were screened based on the titles and abstracts, and 940 of them were assessed for eligibility by reviewing the full texts. Finally, 75 RCTs were included in the systematic review.

### Study Characteristics

The characteristics of the included studies are shown in Table S3 in Multimedia Appendix 1 [43-117]. The 75 RCTs had a total of 8636 participants. In one study, participants’ mean age was 16.8 years, and the mean ages of the participants in the remaining studies ranged from 30.4 to 84.7 years. The intervention duration ranged from 10 days to 18 months, with 44 (59%) studies having 3 to 6 months of intervention. The participants in 10 (13%) studies were depressed at baseline, in accordance with these studies’ inclusion criteria; the participants in 5 studies were diagnosed with depression, and the participants in 5 studies screened positive for depressive symptoms based on scales. No study was on patients diagnosed with anxiety. The sample sizes ranged from 17 to 1023 participants, with 25 (33%) studies having a sample size of ≥100. A total of 68 studies reported depressive symptoms as the outcome, 39 studies reported anxiety symptoms, and 32 studies reported both.

**Figure 2.**
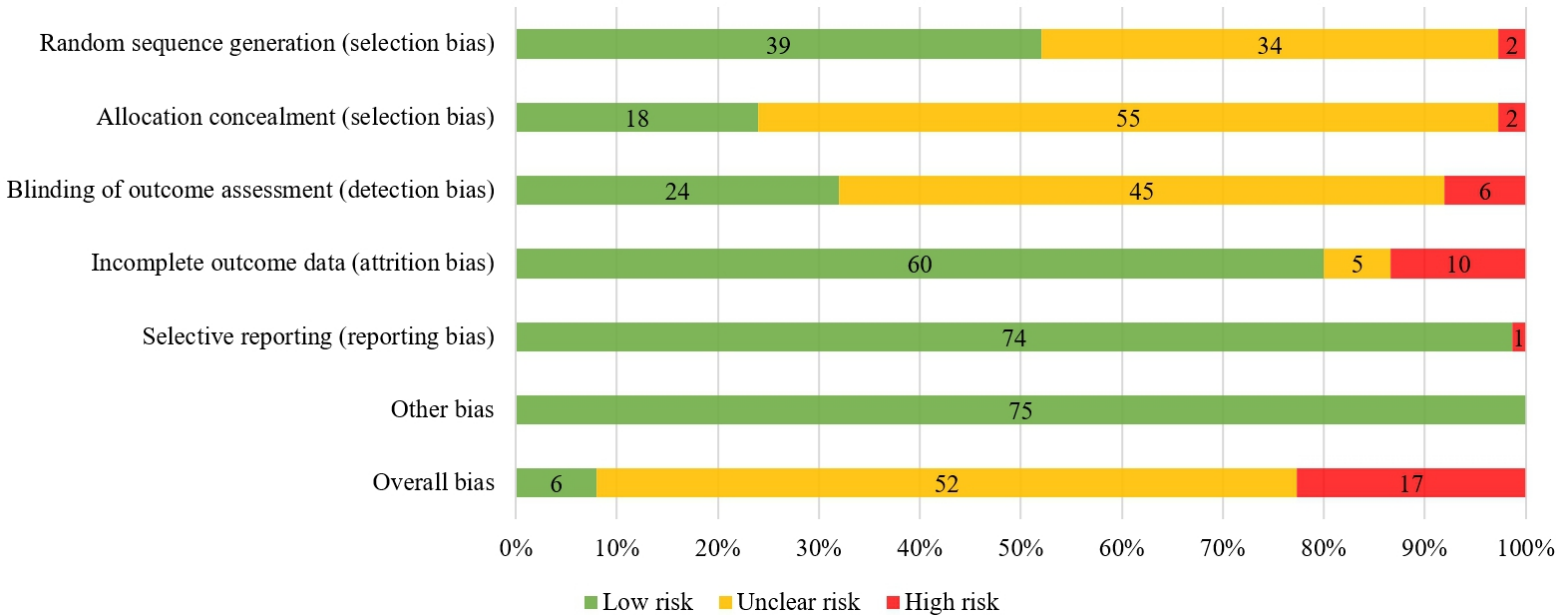
Summary of risk of bias for studies assessing the effect of walking on symptoms of depression and anxiety. Risk of bias was evaluated by using the Cochrane Risk of Bias tool.

The risk of bias in each study is shown in Table S4 in Multimedia Appendix 1 [43-117] and is summarized in Figure 2. The risk of bias was low for random sequence generation in 39 (52%) studies, allocation concealment in 18 (24%) studies, blinding of the outcome assessment in 24 (32%) studies, incomplete outcome data in 60 (80%) studies, selective reporting in 74 (99%) studies, and other bias in 75 (100%) studies. Overall, 6 (8%) studies had a low risk of bias, 52 (69%) studies had an unclear risk of bias, and 17 (23%) had a high risk of bias.

### Primary Analysis: Walking Versus Inactive Controls

Overall, 45 studies compared walking with inactive controls in terms of reducing depressive symptoms. One study with adolescents was not included in the meta-analysis; it reported that walking significantly reduced depressive symptoms in female high school students with depression [43]. The studies included in each subgroup in the subgroup analysis are summarized in Table S5 in Multimedia Appendix 1 [46-51,60-67,71,73-76,78,80,81,83-85,87,88,90,91,93-95,97-104,106,108,110-112,114-116].

**Figure 3.**
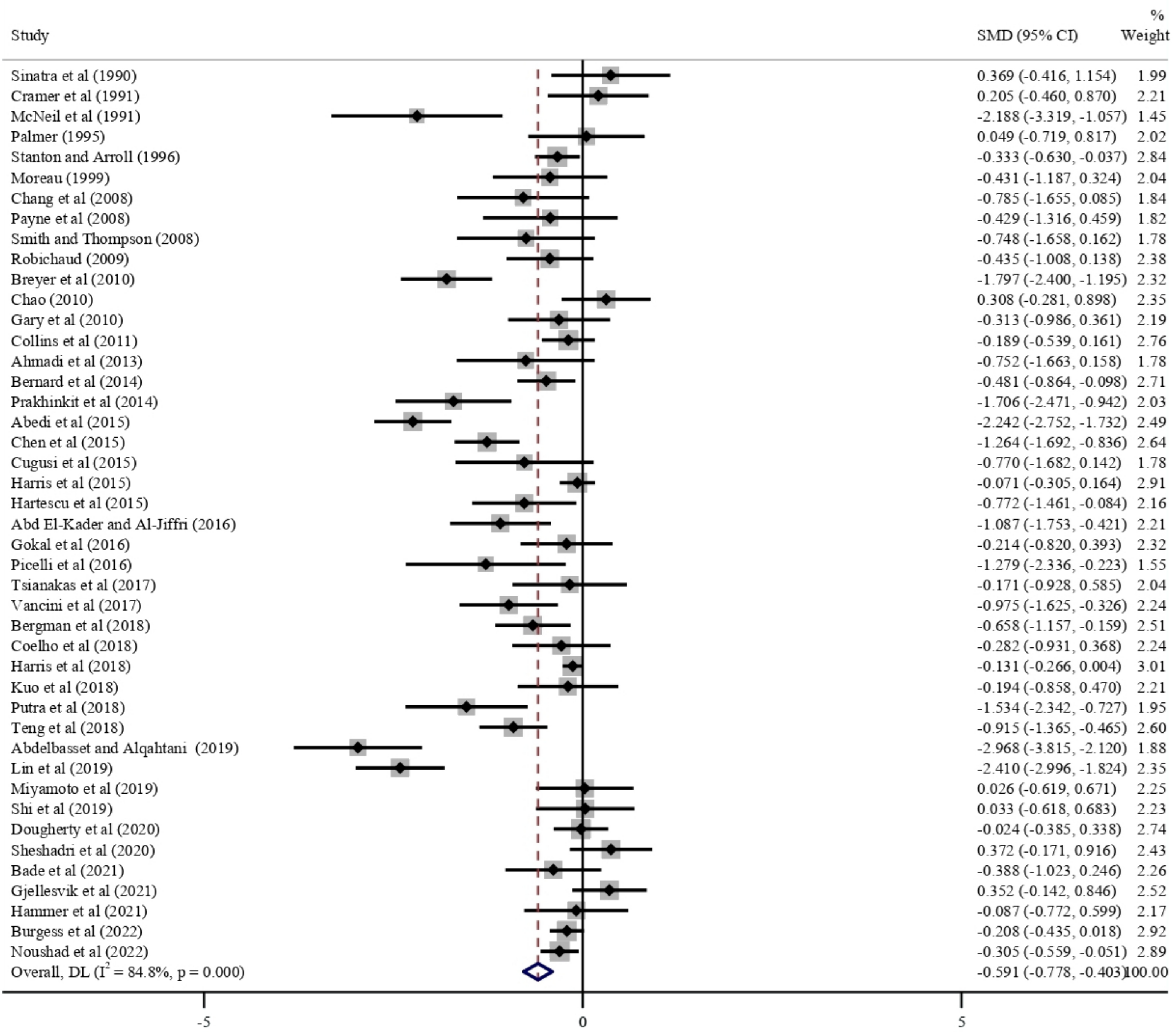
Forest plot of the meta-analysis for the effect of walking on depressive symptoms compared with that of inactive controls [46-51,60-67,71,75,76,78,80,81,83-85,87,88,90,91,93-95,97-102,104,106,108,110-112,114,116]. DL: DerSimonian–Laird; SMD: standardized mean difference.

The pooled results of the remaining 44 studies on adults indicated that walking could significantly reduce depressive symptoms (SMD −0.591, 95% CI −0.778 to −0.403; *I*^2^=84.8%; τ^2^=0.3008; *P*<.001; Table 2). In all the walking subgroups, except walking at a self-selected pace and group walking, walking could significantly reduce depressive symptoms when compared with the inactive controls (all *P* values were <.05). In the subgroup analysis, the intervention effects were significantly better in participants who were depressed (SMD −1.863, 95% CI −2.764 to −0.962) than in participants who were not depressed (SMD −0.442, 95% CI −0.604 to −0.280; *P*=.002), in interventions with a low dropout rate of 0% to 10% (SMD −0.779, 95% CI −1.086 to −0.471) than in those with a dropout rate of >10% (SMD −0.399, 95% CI −0.594 to −0.204; *P*=.04), in participants whose mean age was >60 years (SMD −0.885, 95% CI −1.261 to −0.509) than in those whose mean age was 30 to 60 years (SMD −0.417, 95% CI −0.659 to −0.176; *P*=.04), in interventions without motivation (SMD −0.763, 95% CI −1.045 to −0.480) than in those with motivation (SMD −0.292, 95% CI −0.476 to −0.107; *P*=.006), and in interventions with no pedometers (SMD −0.766, 95% CI −1.032 to −0.500) than in those with pedometers (SMD −0.292, 95% CI −0.532 to −0.052; *P*=.01). The forest plots are shown in Figure 3. In the multivariate meta-regression (*R*^2^=37.3%; Table S6 in Multimedia Appendix 1), baseline depression was significantly associated with a greater decline in depressive symptoms (SMD −1.158, 95% CI −1.851 to −0.466; *P*=.001).

**Figure 4.**
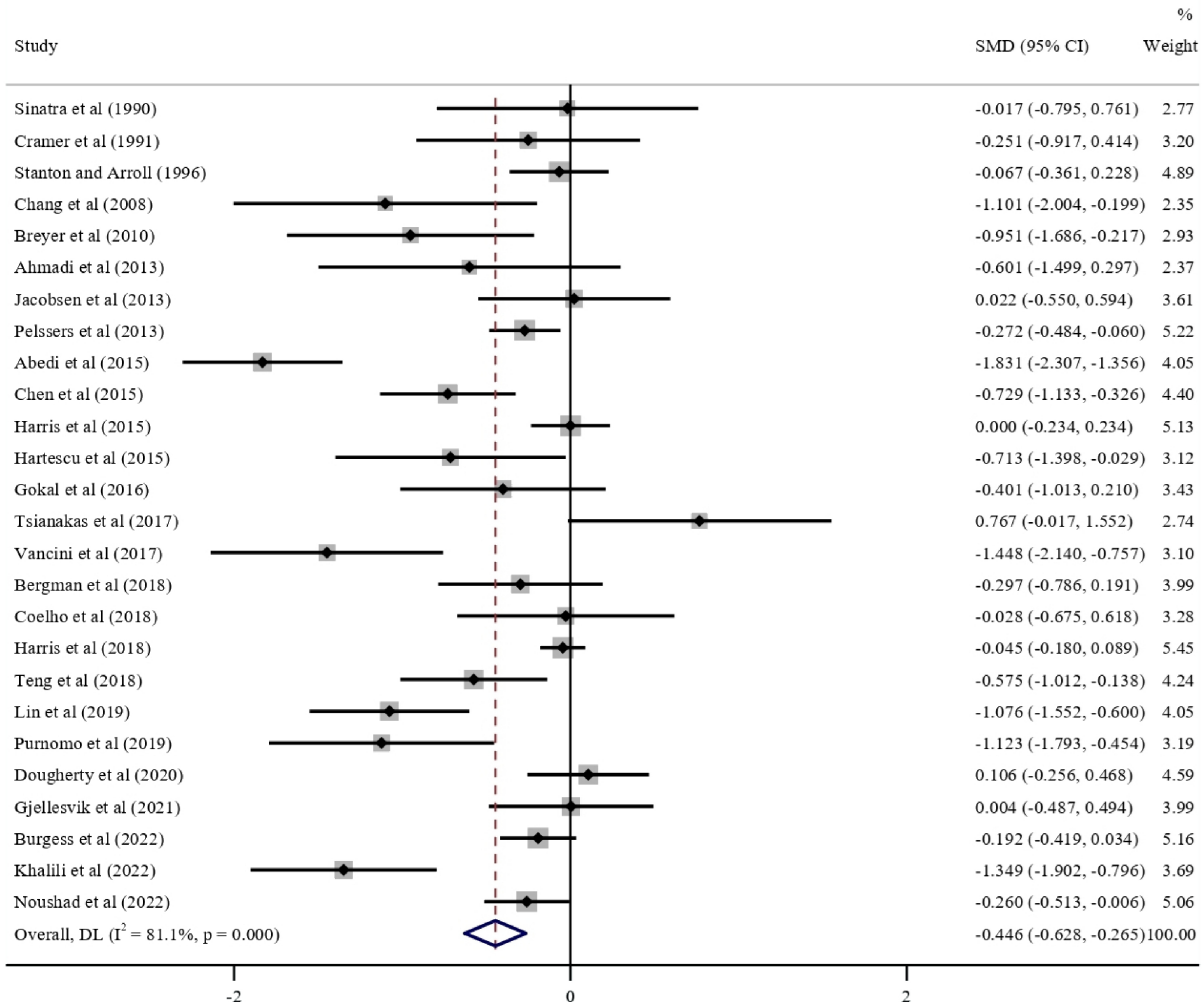
Forest plot of the meta-analysis for the effect of walking on anxiety symptoms compared with that of inactive controls [46,47,50,60,64,71,73,74,78,80,83,84,87,90,91,93-95,99,101,103,106,111,114-116]. DL: DerSimonian–Laird; SMD: standardized mean difference.

The pooled results of 26 studies on adults indicated that walking had significant positive effects on anxiety symptoms when compared with inactive controls (SMD −0.446, 95% CI −0.628 to −0.265; *I*^2^=81.1%; τ^2^=1.530; *P*<.001; Table 3). In all the walking subgroups, except walking at a self-selected pace and motivation, walking significantly reduced anxiety symptoms when compared with inactive controls (all *P* values were <.05). In the subgroup analysis, intervention effects were significantly better in interventions with a duration of <3 months (SMD −0.837, 95% CI −1.255 to −0.418) than in those with a duration of 3 to 6 months (SMD −0.312, 95% CI −0.538 to −0.085; *P*=.03), in interventions involving following instructions during walking (SMD −0.691, 95% CI −0.992 to −0.391) than in those not involving following instructions (SMD −0.273, 95% CI −0.488 to −0.057; *P*=.03), and in interventions without motivation (SMD −0.658, 95% CI −0.932 to −0.384) than in those with motivation (SMD −0.153, 95% CI −0.320 to 0.014; *P*=.002). The forest plots are shown in Figure 4. In the multivariate meta-regression (Table S6 in Multimedia Appendix 1), no factor was significantly associated with anxiety symptoms (*R*^2^=17%; all *P* values were >.05).

The funnel plots are presented in Figure S1 in Multimedia Appendix 1 and are visually and statistically significantly asymmetrical (depressive symptoms: Egger test *t*=−2.02; *P*=.005; anxiety symptoms: Egger test *t*=−2.12; *P*=.01), which indicated the existence of small-study effects.

**Table 2.** The effect of walking (all and different forms) on depressive symptoms compared with that of inactive controls^a^ in the main meta-analysis and subgroup analyses.

Walking intervention, participant, and study characteristics		Studies, n	SMD^b^ (95% CI)	*I*^2^, %	τ^2^	*P* value^c^
All forms of walking		44	−0.591 (−0.778 to −0.403)	84.8	0.3008	—^d^
**Intervention duration (mo)^e^**						.45
	<3	15	−0.743 (−1.199 to −0.287)	83.7	0.6614	
	3‐6	26	−0.545 (−0.778 to −0.323)	86.5	0.2818	
**Intensity**						—
	At least moderate intensity	22	−0.485 (−0.719 to −0.252)	82.2	0.2217	
	Increasing intensity	22	−0.458 (−0.699 to −0.217)	80.6	0.2238	
**Frequency (number of times/wk)^e^**						.29
	<5	26	−0.700 (−0.971 to −0.429)	79.6	0.3674	
	≥5	12	−0.472 (−0.791 to −0.153)	85.9	0.2442	
**Duration of each walk (min)^e^**						.65
	10‐30	18	−0.621 (−0.908 to −0.334)	82.1	0.2701	
	35‐60	18	−0.718 (−1.012 to −0.423)	83.3	0.3072	
**Walking pace^e^**						.52
	Self-selected pace	7	−0.415 (−1.037 to 0.206)	91.6	0.6314	
	Guided or set pace	31	−0.630 (−0.840 to −0.420)	81	0.2462	
**Walking format^e^**						.95
	Individual	18	−0.423 (−0.658 to −0.188)	83.9	0.1892	
	Group	5	−0.440 (−0.947 to 0.068)	60.7	0.1964	
**Walking location^e^**						.81
	Outdoor	8	−0.765 (−1.198 to −0.333)	79.2	0.2787	
	Indoor	14	−0.839 (−1.268 to −0.410)	81.4	0.5280	
**Following instructions during walking**						.06
	Yes	22	−0.785 (−1.108 to −0.461)	85.5	0.4825	
	No	22	−0.411 (−0.634 to −0.189)	81.9	0.1946	
**Walking training**						.90
	Yes	14	−0.611 (−0.961 to −0.261)	86.6	0.3545	
	No	30	−0.583 (−0.815 to −0.351)	84.2	0.3120	
**Motivation (behavior change technique)** ^f^						.006
	Yes	16	−0.292 (−0.476 to −0.107)	70.1	0.0836	
	No	29	−0.763 (−1.045 to −0.480)	87.4	0.4774	
**Pedometer**						.01
	Yes	15	−0.292 (−0.532 to −0.052)	81.6	0.1547	
	No	29	−0.766 (−1.032 to −0.500)	83.1	0.4119	
**Dropout rate (%)**						.04
	0‐10	25	−0.779 (−1.086 to −0.471)	89.9	0.4968	
	>10	17	−0.399 (−0.594 to −0.204)	59.7	0.0883	
**Sample size, n**						.42
	<100	34	−0.638 (−0.908 to −0.367)	81.3	0.5126	
	≥100	10	−0.479 (−0.753 to −0.204)	89.9	0.1688	
**Mean age (y)^e^**						.04
	30‐60	26	−0.417 (−0.659 to −0.176)	82	0.2963	
	>60	16	−0.885 (−1.261 to −0.509)	86.3	0.4682	
**Baseline depressive symptoms**						.002
	Depressed	5	−1.863 (−2.764 to −0.962)	86.4	0.8929	
	Nondepressed	39	−0.442 (−0.604 to −0.280)	77.5	0.1742	
**Risk of bias**						.75
	Low or unclear	34	−0.610 (−0.848 to −0.371)	82.4	0.3861	
	High	10	−0.544 (−0.879 to −0.209)	89	0.2271	

a Inactive controls included no intervention, waitlists, usual care, brief instructions on physical activity, and placebo meetings.

b SMD: standardized mean difference (a higher SMD means more depressive symptoms).

c Between-group difference.

d Not applicable.

e Studies with missing data on the specific subgroup were excluded from the corresponding subgroup analysis.

f Two arms in one study can be categorized into two different subgroups.

**Table 3.** The effect of walking (all and different forms) on anxiety symptoms compared with that of inactive controls^a^ in the main meta-analysis and subgroup analyses.

Walking intervention, participant, and study characteristics		Studies, n	SMD^b^ (95% CI)	*I*^2^, %	τ^2^	*P* value^c^
All forms of walking		26	−0.446 (−0.628 to −0.265)	81.1	0.1530	—^d^
**Intervention duration (mo)^e^**						.03
	<3	8	−0.837 (−1.255 to −0.418)	80	0.2664	
	3‐6	16	−0.312 (−0.538 to −0.085)	81.2	0.1478	
**Intensity**						—
	At least moderate intensity	14	−0.317 (−0.523 to −0.111)	70.8	0.0897	
	Increasing intensity	10	−0.387 (−0.690 to −0.084)	86.1	0.1727	
**Frequency (number of times/wk)^e^**						.55
	<5	12	−0.471 (−0.778 to −0.164)	77.9	0.2048	
	≥5	11	−0.355 (−0.576 to −0.135)	67.4	0.0774	
**Duration of each walk (min)^e^**						.92
	10‐30	14	−0.432 (−0.674 to −0.190)	79	0.1360	
	35‐60	8	−0.411 (−0.700 to −0.122)	72.4	0.1151	
**Walking pace^e^**						.56
	Self-selected pace	4	−0.625 (−1.459 to 0.208)	93.5	0.6668	
	Guided or set pace	17	−0.374 (−0.552 to −0.195)	68.5	0.0765	
**Walking format^e^**						.41
	Individual	13	−0.389 (−0.639 to −0.138)	84.8	0.1590	
	Group	5	−0.588 (−0.990 to −0.187)	70.5	0.1347	
**Walking location^e^**						.09
	Outdoor	5	−0.991 (−1.567 to −0.414)	82.5	0.3419	
	Indoor	5	−0.416 (−0.733 to −0.099)	32.2	0.0413	
**Following instructions during walking**						.03
	Yes	12	−0.691 (−0.992 to −0.391)	71.9	0.1872	
	No	14	−0.273 (-0.488 to −0.057)	82	0.1177	
**Walking training**						.67
	Yes	8	−0.393 (−0.682 to −0.103)	73.7	0.1143	
	No	18	−0.475 (−0.714 to −0.236)	83.9	0.1919	
**Motivation (behavior change technique)** ^f^						.002
	Yes	11	−0.153 (−0.320 to 0.014)	57.1	0.0384	
	No	16	−0.658 (−0.932 to −0.384)	85.3	0.2296	
**Pedometer**						.17
	Yes	10	−0.301 (−0.555 to −0.047)	84.7	0.1228	
	No	16	−0.554 (−0.810 to −0.298)	74.1	0.1830	
**Dropout rate (%)^e^**						.42
	0‐10	11	−0.542 (−0.860 to −0.224)	87.2	0.2186	
	>10	13	−0.381 (−0.615 to −0.146)	71.7	0.1151	
**Sample size, n**						.12
	<100	16	−0.576 (−0.853 to −0.299)	67.9	0.2094	
	≥100	10	−0.294 (−0.520 to −0.067)	86	0.1068	
**Mean age (y)^e^**						.76
	30‐60	18	−0.452 (−0.688 to −0.216)	77.2	0.1801	
	>60	7	−0.523 (−0.929 to −0.118)	86.5	0.2366	
**Risk of bias**						.72
	Low or unclear	19	−0.423 (−0.620 to −0.226)	65.1	0.1070	
	High	7	−0.502 (−0.881 to −0.124)	92	0.2268	

a Inactive controls included no intervention, waitlists, usual care, brief instructions on physical activity, and placebo meetings.

b SMD: standardized mean difference (a higher SMD means more depressive symptoms).

c Between-group difference.

d Not applicable.

e Studies with missing data on the specific subgroup were excluded from the corresponding subgroup analysis.

f Two arms in one study can be categorized into two different subgroups.

### Secondary Analysis: Walking Versus Active Controls or Other Controls

The effects of walking on depressive and anxiety symptoms compared with those of active controls are summarized as forest plots in Figures S2 and S3 in Multimedia Appendix 1 [44,45,48,54,55,59,66,68,70-72,77,82,89,91,92,103,105,107,109,113,117]. There was no significant difference between walking and active controls in reducing depressive symptoms (RCTs: n=17; SMD −0.126, 95% CI −0.343 to 0.092; *I*^2^=58%; τ^2^=0.1058; *P*=.26) and anxiety symptoms (RCTs: n=14; SMD −0.053, 95% CI −0.311 to 0.206; *I*^2^=67.7%; τ^2^=0.1421; *P*=.69). The funnel plots are presented in Figure S4 in Multimedia Appendix 1. The Egger test showed no significant small-study effects for depressive symptoms (*t*=−1.02; *P*=.28) and anxiety symptoms (*t*=−0.60; *P*=.57).

The effects of walking on depressive symptoms were compared to those of light exercise, nutrition, and regular education in 6, 3, and 6 studies, respectively (Figure S5 in Multimedia Appendix 1 [44,46,52-54,56-58,69,79,86,96,98,105]). Walking had statistically significantly better effects on depressive symptoms than light exercise (SMD −0.426, 95% CI −0.766 to −0.086; *I*^2^=55.9%; τ^2^=0.1148; *P*=.046). There was no statistical difference when comparing walking with nutrition (SMD −0.091, 95% CI −0.541 to 0.358; *I*^2^=50.2%; τ^2^=0.0795; *P*=.69) and with regular education (SMD −0.208, 95% CI −0.476 to 0.060; *I*^2^=59.6%; τ^2^=0.0622; *P*=.13) in terms of depressive symptoms. There was no statistical difference between 4 studies that compared walking with light exercise in terms of anxiety symptoms (SMD −0.493, 95% CI −1.056 to 0.070; *I*^2^=65.1%; τ^2^=0.1611; *P*=.09).

## Discussion

### Principal Results

This systematic review of 75 studies assessed the effect of walking on depressive and anxiety symptoms and included comprehensive subgroup analyses, inactive controls, and active controls. The results indicated that walking could reduce depressive and anxiety symptoms in adults when compared with the inactive controls. The new findings in this review were that most of the walking subgroups had statistically significant beneficial effects, including different walking frequency, duration, location (indoor or outdoor), and format (group or individual) subgroups, and participants, regardless of whether they were depressed at baseline, benefited from the effects of walking. The effect of walking was also comparable to that of active controls.

### Comparison With Prior Work

The results of the primary analysis, which showed that walking had an overall positive effect on remitted depressive and anxiety symptoms when compared with inactive controls, were consistent with previous meta-analyses on walking [22,23] and comparable to a meta-analysis of physical activity interventions [118]. However, our study did not find a dose-response relationship between walking, including walking frequency and the duration of each walk, and symptoms of depression or anxiety. In a recent meta-analysis of prospective cohort studies, the activity volume equivalents of 1.25 and 2.5 hours of brisk walking per week, which are shorter than the recommendation of 150 minutes of moderate-intensity physical activity per week from the World Health Organization (WHO) [119], were respectively associated with an 18% and 25% lower risk of depression [120]. These findings indicate that a shorter walking duration could still result in significant mental health benefits, albeit longer walks are better [121,122].

In most of the subgroup analyses, there was no statistical difference between the subgroups (all *P* values were >.05). This study found that both outdoor walking and indoor walking were effective in alleviating depression and anxiety, which is consistent with a previous meta-analysis on walking from 2012 [22]. It suggested that the effect of walking has no association with the location of walking (ie, indoors or outdoors). This might be important to homebound people (eg, older people or people who were home monitoring during the COVID-19 pandemic). Walking may also be effective regardless of whether people wear a pedometer and how old they are. In this review, walking at a self-selected pace was not effective on the selected mental health symptoms, whereas at least moderate-intensity walking and walking at a guided pace were effective. However, this study did not conduct a subgroup analysis of mild-intensity walking due to the limited number of relevant studies. These findings may indicate that walking at a guided or set pace (eg, a pace that can reach a certain speed or result in a certain heart rate), especially when such walking can reach a moderate intensity, is recommended, which is consistent with the WHO guideline that recommends moderate-intensity physical activity [119]. Future studies can closely examine the effects of mild-intensity walking on mental health.

In the remaining subgroup analyses, both adult participants who were depressed and those who were not depressed could benefit from walking, and participants who were depressed could benefit more. The meta-regression showed that baseline depression was the main factor that affected the effect of walking. The subgroup analysis of baseline anxiety was not conducted on anxiety symptoms due to a lack of data. Shorter intervention durations (<3 mo), walking without applying motivation, and walking without a pedometer were found to be significantly better than intervention durations of 3 to 6 months, walking while applying motivation, and walking with a pedometer, respectively. However, the latter three characteristics also had small and statistically significant positive effects on depressive or anxiety symptoms. The effects might have been confounded by other characteristics of walking interventions (eg, the intensity and frequency of walking), which may have contributed to the effectiveness of walking in the studies on the latter three characteristics. The lower adherence in these studies might also explain the smaller effects. A study found that adherence to physical exercise tends to decrease over time [123]. In addition, most of the walking interventions that applied motivation or involved a pedometer were unsupervised interventions that did not provide regular walking sessions. Supervised walking may be more likely to keep people walking regularly over longer time periods. Adherence to the prescribed dose of physical activity might be worse in unsupervised interventions, which rely on self-report measures to indicate adherence [124]. Better adherence to interventions was found to be associated with larger effects [125]. In this study, adherence was not analyzed in the subgroup analysis because the definition of adherence was different among studies. However, the larger effects in the studies with a low dropout rate of no more than 10% may support the beneficial effects of adherence to walking interventions.

In subgroup analyses, following or providing instructions during walking, such as in supervised walking or by including warm-up and cooldown components, was significantly better for anxiety symptoms than not following or providing instructions during walking. During this kind of walking, a certain walking pace might be settled. In addition, when following instructions, working memory has been shown to increase [126], and working memory training could reduce anxiety and depression vulnerability [127]. Hence, the settlement of walking pace and the involvement of working memory may serve as the moderators in the effect of walking instructions. Further studies are needed to confirm this mechanism.

The effect of walking was found to be comparable to that of active controls, which included other kinds of moderate-intensity physical exercise and other evidence-based interventions. This finding is consistent with previously reported results comparing the effects between physical exercise and other active controls. Exercise has been found to have a small but statistically nonsignificant effect when compared with psychological treatments or antidepressant medication [128]. However, there is no study comparing several different kinds of physical exercises (eg, via a network meta-analysis), including walking, in terms of their effects on mental health outcomes. Based on the existing evidence, walking (especially at a pace that can reach a certain speed, result in a certain heart rate, or reach moderate intensity) can be recommended as an alternative to other exercises and evidence-based interventions to improve mental health outcomes. Future studies can explore the dose-response relationship between walking and reduced mental health symptoms to provide more detailed instructions to people in need.

Based on the results found in this systematic review, incorporating walking into public health policies and initiatives can potentially decrease the prevalence of depression and enhance overall mental well-being. The results can be used to support mental health services in community and clinical settings that may incorporate walking as part of their prevention and treatment plans. Incorporating walking into indoor workplace wellness programs may improve employee mental health and productivity. Furthermore, health care care professionals should encourage and recommend at least moderate-intensity walking and such walking at a guided pace to the population to reduce depressive symptoms. Walking promotion should be included in the training and education for health care professionals and other health-related workers. They should recommend walking regularly to people in need, as well as supervise regular walking sessions, to ensure adherence to the walking exercise, and when resources are insufficient, priority should be given to people who are depressed.

### Strengths and Limitations

The strengths of this systematic review and meta-analysis are that it included a large number of studies and confirmed the beneficial effects of walking on symptoms of depression and anxiety. A comprehensive subgroup analysis was conducted to explore the effects of different forms of walking. The effect of walking was also compared with that of different controls based on evidence, including inactive controls, active controls, and other controls.

This study has a few limitations that should be acknowledged. First, there was a high level of between-study heterogeneity in the primary analysis, although a subgroup analysis was performed to explore the potential reason for heterogeneity. Therefore, the results should be treated with caution when interpreting them and generalizing them to the real-world setting. Second, there existed small-study effects in the primary analysis, which could have been due to multiple reasons, such as clinical heterogeneity, outcome reporting bias, publication bias, and coincidence [129]. Third, most of the studies had an unclear (52/75, 69%) or high (17/75, 23%) risk of bias. This was due to the lack of reporting on allocation concealment and blinding of the outcome assessment. However, this is common among published RCTs. Previous literature suggested that only 6% of trials could be assessed as having an overall low risk of bias [130]. In this meta-analysis, a subgroup analysis was conducted to exclude the impact of studies with a high risk of bias and found that there was no significant difference between those with a high risk of bias and the remaining studies. Fourth, this study only measured the postintervention effect size. Further studies can be performed to measure the effects of the long-term maintenance of regular walking during follow-ups. In addition, as mentioned in the *Comparison With Prior Work* section, there is a lack of studies on mild-intensity walking. Future studies shall take a closer look at the effects of mild-intensity walking, besides the dose-response effects of walking on depression and anxiety. Fifth, most of the participants included in this meta-analysis did not have a diagnosis of depression or anxiety disorder. More walking interventions designed for diagnosed patients can be conducted in the future. Last, there is a lack of relevant trials on children and adolescents, which can be a future direction for research.

### Conclusions

In summary, walking is an effective and promising intervention for reducing symptoms of depression and anxiety when compared with inactive controls. Its effects are comparable to active controls. Various forms of walking can serve as choices of treatment for people with symptoms of depression and anxiety. Integrating walking (especially at a pace that can reach moderate intensity) into public health initiatives can have potential benefits in reducing depression and anxiety. Future studies can explore the dose-response and long-term effects of walking on depression and anxiety.

## Supplementary material

10.2196/48355 Multimedia Appendix 1 Search strategies, subgroup definitions, characteristics of included studies, the risk of bias of included trials, subgroup analysis results, meta-regression results, funnel plots, and forest plots.

10.2196/48355 Checklist 1 PRISMA (Preferred Reporting Items for Systematic Reviews and Meta-Analyses) checklist.

## Abbreviations

AMED: Allied and Complementary Medicine Database
CENTRAL: Cochrane Central Register of Controlled Trials
PRISMA: Preferred Reporting Items for Systematic Reviews and Meta-Analyses
PROSPERO: International Prospective Register of Systematic Reviews
RCT: randomized controlled trial
SMD: standardized mean difference
WHO: World Health Organization
