# Supplementary material for: The Effect of Walking on Depressive and Anxiety Symptoms: Systematic Review and Meta-Analysis
Source: JMIR Public Health Surveill. 2024 Jul 23;10:e48355. doi: 10.2196/48355 (PMC11287235; doi:10.2196/48355)
Supplement: Multimedia Appendix 1 [file publichealth-v10-e48355-s001.docx]

**Table S1.** Search strategy of the seven databases

| Database | Search strategies | Records |
| --- | --- | --- |
| MEDLINE  (Pubmed) | #1 walking[MeSH Terms] OR walk*[All Fields] OR pedometer*[All Fields] OR "step count*"[All Fields]  #2 depression[MeSH Terms] OR depressive disorder[MeSH Terms] OR depress*[All Fields]  #3 mood disorders[MeSH Terms] OR dysthymia[All Fields] OR dysthymic[All Fields] OR "mood disorders"[All Fields] OR "mood disorder"[All Fields] OR "affective disorder"[All Fields] OR "affective disorders"[All Fields]  #4 anxiety[MeSH Terms] OR anxiety disorders[MeSH Terms] OR anxiety[All Fields] OR anxious[All Fields] OR "anxiety disorders"[All Fields] OR "anxiety disorder"[All Fields]  #5 fear[MeSH Terms] OR "phobic disorders"[All Fields] OR phobia[All Fields] OR phobias[All Fields] OR fear[All Fields] OR "panic disorder"[All Fields] OR "panic attack"[All Fields] OR agoraphobia[All Fields] OR agoraphobias[All Fields] OR obsessive[All Fields] OR compulsive[All Fields] OR OCD[All Fields] OR GAD[All Fields]  OR PTSD[All Fields] OR posttrauma[All Fields] OR post-trauma[All Fields] OR "stress disorder"[All Fields] OR "stress disorders"[All Fields]  #6 #2 OR #3 OR #4 OR #5  #7 randomized controlled trials as topic[MeSH Terms] OR controlled clinical trials as topic[MeSH Terms] OR clinical trials as topic[MeSH Terms] OR randomized controlled trial[Publication Type] OR controlled clinical trial[Publication Type] OR "randomized controlled trial"[All Fields] OR "randomised controlled trial"[All Fields] OR "controlled clinical trial"[All Fields] OR random*[Title/Abstract] OR placebo[Title/Abstract] OR trial[Title/Abstract] OR sham[Title/Abstract]  #8 #1 AND #6 AND #7 | 2497 |
| The Cochrane Central Register of Controlled Trials (CENTRAL) | #1 MeSH descriptor: [Walking] explode all trees  #2 walk* or pedometer* or (step NEXT count*) or (count* NEXT of NEXT step*)  #3 #1 or #2  #4 MeSH descriptor: [Depression] explode all trees  #5 MeSH descriptor: [Mood Disorders] explode all trees  #6 depress* or dysthymi* or (mood NEXT disorder*) or (affective NEXT disorder*) or (affective NEXT symptom*)  #7 MeSH descriptor: [Anxiety] explode all trees  #8 MeSH descriptor: [Anxiety Disorders] explode all trees  #9 anxiety or anxio* or phobi* or fear* or (panic NEXT disorder*) or (panic NEXT attack) or agoraphobi* or obsessi* or compulsi* or OCD or GAD or PTSD or posttrauma* or post-trauma* or (post NEXT trauma*) or (stress NEXT disorder*)  #10 #4 or #5 or #6 or #7 or #8 or #9  #11 #3 and #10 (Content type-Trials) | 4401 |
| OVID EMBASE | 1. exp walking/  2. (walk* or pedometer* or step count* or count of step*).tw.  3. 1 or 2  4. exp depression/  5. (depress* or dysthymi*).tw.  6. (mood disorder* or affective disorder* or affective symptom*).mp.  7. exp anxiety/  8. exp anxiety disorder/  9. (anxiety or anxio* or phobi* or fear* or panic disorder* or panic attack or agoraphobi* or obsessi* or compulsi* or OCD or GAD or PTSD or posttrauma* or post-trauma* or post trauma* or stress disorder*).tw.  10. 4 or 5 or 6 or 7 or 8 or 9  11. randomized controlled trial.de.  12. randomization.de.  13. randomi#ed.ti,ab.  14. randomly.ab.  15. trial*.ab.  16. placebo.ab.  17. sham.ab.  18. (control* adj3 (trial* or study or studies or group*)).ab,ti.  19. 11 or 12 or 13 or 14 or 15 or 16 or 17 or 18  20. 3 and 10 and 19 | 4039 |
| OVID PsycINFO | 1. exp walking/  2. (walk* or pedometer* or step count* or count of step*).tw.  3. 1 or 2  4. exp depression/  5. exp major depression/  6. exp atypical depression/  7. (depress* or dysthymi*).tw.  8. (mood disorder* or affective disorder* or affective symptom*).mp.  9. exp anxiety/  10. exp anxiety disorder/  11. exp panic/  12. exp panic attack/  13. (anxiety or anxio* or phobi* or fear* or panic disorder* or panic attack or agoraphobi* or obsessi* or compulsi* or OCD or GAD or PTSD or posttrauma* or post-trauma* or post trauma* or stress disorder*).tw.  14. 4 or 5 or 6 or 7 or 8 or 9 or 10 or 11 or 12 or 13  15. treatment effectiveness evaluation.sh.  16. clinical trials.sh.  17. mental health program evaluation.sh.  18. placebo.sh.  19. placebo.ab.  20. randomly.ab.  21. randomi#ed.ti,ab.  22. sham.ab.  23. trial*.ti,ab.  24. (control* adj3 (trial* or study or studies or group*)).ti,ab.  25. 15 or 16 or 17 or 18 or 19 or 20 or 21 or 22 or 23 or 24  26. 3 and 14 and 25 | 557 |
| OVID AMED | 1. exp walking/  2. (walk* or pedometer* or step count* or count of step*).tw.  3. 1 or 2  4. exp depression/  5. exp depressive disorder/  6. (depress* or dysthymi*).tw.  7. (mood disorder* or affective disorder* or affective symptom*).mp.  8. exp anxiety/  9. exp anxiety disorder/  10. exp panic/  11. (anxiety or anxio* or phobi* or fear* or panic disorder* or panic attack or agoraphobi* or obsessi* or compulsi* or OCD or GAD or PTSD or posttrauma* or post-trauma* or post trauma* or stress disorder*).tw.  12. 4 or 5 or 6 or 7 or 8 or 9 or 10 or 11  13. exp clinical trials/  14. randomized controlled trial.pt.  15. exp comparative study/  16. exp random allocation/  17. exp placebos/  18. placebo.ab.  19. randomly.ab.  20. randomi#ed.ti,ab.  21. sham.ab.  22. trial*.ti,ab.  23. (control* adj3 (trial* or study or studies or group*)).ti,ab.  24. 13 or 14 or 15 or 16 or 17 or 18 or 19 or 20 or 21 or 22 or 23  25. 3 and 12 and 24 | 159 |
| CINAHL (EBSCOhost) | S1 TX (walk* OR pedometer* OR "step count*" or "count of step*")  S2 MH walking+  S3 S1 OR S2  S4 MH "affective disorders+"  S5 TX (depress* OR dysthymi*)  S6 TX ("mood disorder*" OR "affective disorder*" OR "affective symptom*")  S7 MH anxiety+ OR MH anxiety disorders+  S8 TX (anxiety OR anxio* OR phobi* OR fear* OR "panic disorder*" OR "panic attack" OR agoraphobi* OR obsessi* OR compulsi* OR OCD OR GAD OR PTSD OR posttrauma* OR post-trauma* OR "post trauma*" OR "stress disorder*")  S9 S4 OR S5 OR S6 OR S7 OR S8  S10 PT "clinical trial"  S11 MH "clinical trials+" OR MH "random assignment+"  S12 AB (placebo OR randomly OR randomi#ed OR sham OR trial*)  S13 AB (control* N3 (trial* OR study OR studies OR group*))  S14 S10 OR S11 OR S12 OR S13  S15 S3 AND S9 AND S14 | 7598 |
| ISI Web of Science | #1 ALL=(walk* OR pedometer* OR "step count*" OR "count of step*")  #2 ALL=(depress* or dysthymi*)  #3 TS=("mood disorder*" or "affective disorder*" or "affective symptom*")  #4 ALL=(anxiety or anxio*)  #5 TS=(phobi* or fear* or "panic disorder*" or "panic attack" or agoraphobi* or obsessi* or compulsi* or OCD or GAD or PTSD or posttrauma* or post-trauma* or "post trauma*" or "stress disorder*")  #6 #2 or #3 or #4 or #5  #7 AB=(placebo or random* or sham or trial*)  #8 AB=(control* adj (trial* or study or studies or group*))  #9 #7 OR #8  #10 #1 and #6 and #9 | 4566 |

**Table S2.** Definition of each subgroup

| Subgroup | Definition |
| --- | --- |
| At least moderate intensity | First, defined by the study itself. If there was no definition on intensity, moderate intensity was defined by at least 50% of maximum heart rate. |
| Increasing intensity | Included increasing walking time, frequency, distance or intensity. |
| Guided/set pace | Supervised pace or a pace that can reach certain speed or heart rate. |
| Following instruction during walking | Supervised walking or walking with specific instructions, e.g. stretching/warm up, walking, and cool down. |
| Walking training | Provided prior training or education on walking. |
| Motivation | Applied behavior change theories or techniques such as motivation, motivational interviewing, addressing barriers, and self-efficacy, etc. |
| Pedometer | Wore pedometer during walking. |
| Dropout rate | (1 – number of participants at follow-up / number of participants at baseline) * 100%. |
| Baseline depressive symptoms | Having depressive symptoms at baseline according to the inclusion criteria of each study. |

|  |  |  |  |  |  |  |
| --- | --- | --- | --- | --- | --- | --- |

**Table S3.** Characteristics of included studies

| Study | Country/  Region | Population | Sample size | Intervention | Intervention duration | Mean age | Female (%) | Depression scale | Anxiety scale |
| --- | --- | --- | --- | --- | --- | --- | --- | --- | --- |
| Minor (1989)^1^ | USA | Patients with rheumatoid arthritis or osteoarthritis | 115 | - Aerobic walking met for 1 hour, 3 times a week (n=36) - Aerobic aquatics met for 1 hour, 3 times a week (n=47) - Range of motion exercise control group met for 1 hour, 3 times a week (n=32) | 12 weeks |  |  | AIMS | AIMS |
| Sexton (1989)^2^ | Norway | Patients with symptomatic neurotics | 53 | - The group walked 30 minutes 3-4 times per week (n=25) - The group jogged 30 minutes 3-4 times per week (n=28) | 8 weeks | - 39.2 - 36.4 | - 60 - 46.4 | BDI | STAI |
| Sinatra (1990)^3^ | USA | Healthy, middle-aged women | 94 | - Placebo meeting with one 60-min session (n=12) - Committed walking with gradually increasing distances with 3 days per week (n=28) - Continuous passive motion exercise 60min/session with 3 days per week (n=25) - Continuous passive motion combined with dieting met once a week for 90min (n=29) | 12 weeks | Overall 42.9 |  | BDI | STAI |
| Cramer (1991)^4^ | USA | Sedentary, mildly obese women | 50 | - Brisk walking five 45 min sessions/week (n=25) - Non-exercise group (n=25) | 15 weeks | - 36 - 32.4 | - 100 - 100 | PMOS | PMOS |
| McNeil (1991)^5^ | Canada | Community-dwelling moderately depressed elderly | 30 | - Experimenter-accompanied exercise in the form of walking increased from 20 min to 40 min with three sessions held each week (two with a psychology student, one alone) - A social contact control with two home visits each week increased from 20 to 40 min - Wait-list control | 6 weeks | Overall 72.5 |  | BDI |  |
| Palmer (1995)^6^ | USA | Nonexercising premenopausal women | 64 | - A supervised walking group with 20 min each session and increased by 5 min each week (n=16) - Waiting-list non-walking group (n=11) | 8 weeks | Overall 37.4 | - 100 - 100 | CES-D |  |
| Stanton (1996)^7^ | New Zealand | Sedentary, mildly hypertensive adults | 208 | - Walking briskly for 40 minutes 3 times per week - Control group | 6 months | Overall 55 | Overall 47.5 | POMS-BI | POMS-BI |
| Moreau (1999)^8^ | USA | Postmenopausal women with borderline to mild hypertension | 52 | - 16 kilometer/week walking group - 32 kilometer/week walking group - Sedentary control group | 12 weeks | - 54.9 - 53.8 - 56.5 |  | CES-D |  |
| Nieman (2000)^9^ | USA | Obese women | 102 | - Control group - Five 45-minute walking sessions/week (4 supervised sessions and one session without supervision) - Diet: moderate energy restriction - Five 45-minute walking sessions/week plus moderate energy restriction | 12 weeks | Overall 45.6 | - 100 - 100 - 100 - 100 | GWBS | GWBS |
| King (2002)^10^ | USA | Postmenopausal female caregivers | 100 | - Home-based, telephone-supervised, moderate-intensity brisk walking training (n=51) - Attention-control (nutrition education) program (n=49) | 12 months | - 62.2 - 63.3 |  | BDI |  |
| Penninx (2002)^11^ | USA | Community-based older adults with knee osteoarthritis | 439 | - Resistance exercise program with a 3-month supervised facility-based program of three 1-hr sessions per week and a 15-month home-based program (n=146) - 3-month facility-based walking program+15-month home-based walking program (n=149) - Monthly health education (n=144) | 3+15 months | Overall 68.8 | Overall 70 | CES-D |  |
| Armstrong (2004)^12^ | Australia | Postnatal women who reported experiencing postnatal depression | 24 | - Pram-walking programme for mothers and their babies for 40 min each session (n=12) - Social support group met once per week (n=12) | 12 weeks |  | - 100 - 100 | EPDS |  |
| Motl (2005)^13^ | USA | Sedentary older adults | 174 | - Walking three times per week increased from 10-15 to 40-45 minutes per session (n=85) - Low-intensity resistance/flexibility training increased from 10-15 to 40-45 minutes per session (n=89) | 6 months | Overall  65.5 | Overall 72% | GDS-30 |  |
| Gary (2006)^14^ | USA | Older women with diastolic heart failure | 32 | - Walking for 3 days per week (n=16) - Education-only with weekly home visits (n=16) | 12 weeks |  |  | GDS-15 |  |
| Knubben (2007)^15^ | Germany | Inpatients with a major depression episode undergoing standard clinical antidepressant drug treatment | 38 | - Daily walking on a treadmill (n=20) - Daily 30-min light stretching exercises and relaxation exercises (n=18) | 10 days | - 49 - 50 | - 55 - 55.6 | BRMS | CES-D |
| Bircan (2008)^16^ | Turkey | Women with fibromyalgia | 30 | - Walking on treadmill increased from 20 to 30 min (n=15) - Strengthening exercise 30 min (n=15) | 8 weeks | - 48.3 - 46 |  | HADS | HADS |
| Chang (2008)^17^ | China (Taiwan) | Hospitalized acute myelogenous leukemia patients undergoing chemotherapy | 24 | - 12 minutes of walking exercise program per day, five days per week (n=12) - Standard ward care (n=12) | 3 weeks | - 49.4 - 53.3 | - 27.3 - 63.6 | PMOS short form | PMOS short form |
| Payne (2008)^18^ | USA | Older women with breast cancer receiving hormonal therapy | 20 | - Moderate walking activity with a pedometer, 20 minutes in duration, four times a week (n=10) - No intervention (n=10) | 14 weeks | Overall 64.7 | - 100 - 100 | CES-D-20 |  |
| Smith (2008)^19^ | USA | People in the chronic stage of recovery from stroke | 20 | - Twelve 20-minute sessions of walking on a treadmill (n=10) - Weekly phone calls (n=10) | 4 weeks | - 57.8 - 56 | - 20 - 60 | BDI |  |
| Robichaud (2009)^20^ | USA | Postpartum women | 51 | - Home-based walking program,30 minutes three times a week (n=27) - Wait-list control group (n=24) | 6 weeks | - 31.1 - 30.4 | - 100 - 100 | EPDS |  |
| Breyer (2010)^21^ | Austria | Patients with chronic obstructive pulmonary disease | 65 | - Nordic Walking three times a week (n=32) - No intervention (n=33) | 3 months | - 61.9 - 59 | - 53 - 57 | HADS | HADS |
| Chao (2010)^22^ | USA | People with severe and persistent mental illness | 60 | - Unsealed pedometer with self-monitoring (n=20) - Sealed pedometer without self-monitoring (n=20) - No pedometer control group (n=20) | 2 weeks | - 46.1 - 48.5 - 46.1 | - 35 - 35 - 30 | CES-D |  |
| Gary (2010)^23^ | USA | Heart failure patients diagnosed with depression | 74 | - Cognitive behavior therapy with 1 hour per session (n=19) - Weekly face-to-face home visits to monitor walking progress and to tailor the exercise prescription, with walking for up to 1 hour per day 3 days per week (n=20) - Usual care (n=17) - Weekly walking and cognitive behavior therapy (n=18) | 12 weeks | Overall 65.8 | Overall 57.1 | HAM-D |  |
| Collins (2011)^24^ | USA | People with diabetes and peripheral arterial disease | 145 | - Home-based walking program with walking training and weekly group walking classes and biweekly telephone calls (n=72) - Attention control group with twice-monthly phone calls (n=73) | 6 months | - 66.2 - 66.8 | - 35 - 27 | GDS-15 |  |
| McCaffrey (2011)^25^ | USA | Older adults with depression | 48 | - Guided group walking for 1 to 2 hours each time (n=14) - Independent walking for 1 to 2 hours each time (n=18) - Art therapy group met with a certified art therapist twice per week (n=16) | 6 weeks | - 74.6 - 73.9 - 74.3 | - 62 - 62 - 54 | GDS-30 |  |
| Roshan (2011)^26^ | Iran | High school female students with depression | 24 | - Pool walking exercise with 3 sessions weekly (n=12) - No intervention (n=12) | 6 weeks | - 16.91 - 16.83 | - 100 - 100 | HAM-D |  |
| Maki (2012)^27^ | Japan | Inhabitants aged 65 and older | 150 | - Community-based walking program with pedometer (n=75) - Educational lectures on food, nutrition, and oral care (n=75) | 12 weeks | - 71.9 - 72 | - 69.3 - 72 | GDS |  |
| Ridsdale (2012)^28^ | UK | People with chronic fatigue | 222 | - Usual care plus cognitive behaviour therapy booklet (n=75) - Graded exercise therapy in the form of walking 5–30 min of exercise a day (n=71) - Counselling (n=76) | 6 months | - 37.3 - 42.6 - 39.7 | - 79 - 79 - 76 | HADS | HADS |
| Ahmadi (2013)^29^ | Iran | Multiple sclerosis patients | 31 | - Supervised treadmill training 30 minutes thrice weekly (n=10) - Yoga 60 - 70 minutes in duration, thrice weekly (n=11) - Control group followed their own routine treatment program (n=10) | 8 weeks | - 36.8 - 32.27 - 36.7 | - 100 - 100 - 100 | BDI | BAI |
| Faulkner (2013)^30^ | New Zealand | Cardiac rehabilitation patients | 22 | - 3 sessions per week, self-paced walking intervention - 30-minute stationary cycling, 3 sessions per week | 4 weeks | - 62.7 - 61.2 | Overall 40.9 |  | Modified HADS |
| Jacobsen (2013)^31^ | USA | Patients undergoing chemotherapy | 460 | - Usual care only with access to the full range of psychosocial services (n=117) - Stress management training (n=114) - Pedometers-based walking with recommendation of 3–5 times per week for 20 to 30 min (n=109) - Stress management training plus exercise (n=120) | 12 weeks | - 57.22 - 57.42 - 58.72 - 57.71 | - 74 - 67 - 61 - 69 | CES-D-20 | BAI-21 |
| Pelssers (2013)^32^ | Belgium | Older adults age 55 and older | 580 | - Pedometer-defined walks in weekly walking schedules, which prescribed walks of a number of aerobic steps (≥10 min) on most to all weekdays (n=432) - Wait-list control condition (n=148) | 10 weeks | - 69.4 - 70.34 | - 69.4 - 62.2 |  | STAI |
| Bernard (2014)^33^ | France | Inactive post-menopausal women without depression | 121 | - Moderate intensity walking intervention (three times a week, 40 minutes per session, supervised and home-based) (n=61) - Waiting list control (n=60) | 6 months | - 65.46 - 65.5 |  | BDI |  |
| Prakhinkit (2014)^34^ | Thailand | Female elderly participants with mild-to-moderate depressive symptoms | 45 | - Sedentary control (n=15) - Traditional walking 3 times a week with increased intensity and time from 20 to 30 min (n=15) - Walking meditation 3 times a week with increased intensity and time from 20 to 30 min (n=15) | 12 weeks | - 81 - 74.8 - 74 | - 100 - 100 - 100 | GDS-30 |  |
| Van Hoecke (2014)^35^ | Belgium | Sedentary older adults | 442 | - A one-contact referral to locally organized physical activities (n=146) - A one-contact advice session in which a Health Fitness Specialist provided a structured walking program in addition to the referral (n=146) - A 10-week multiple-contact individually tailored PA coaching plus the referral and the walking program (n=150) | 10 week | Overall 69.48 | - 68.5 - 64.4 - 67.3 |  | STAI |
| Abedi (2015)^36^ | Iran | Postmenopausal women with mild to moderate depression | 106 | - Pedometers-based walking to increase steps by at least 500 steps per week (n=53) - No intervention (n=53) | 12 weeks | - 52.4 - 53 |  | BDI | 7 questions from GHQ-28 |
| Bellon (2015)^37^ | USA | People with a traumatic brain injury | 123 | - Home-based walking programme with weekly goals and pedometers to track the amount of steps walked daily - Nutrition programme about eating healthy | 12 +12 weeks | Overall 43.7 | Overall 41.8 | CES-D |  |
| Chen (2015)^38^ | China (Taiwan) | Patients with lung cancer | 116 | - Home-based, moderate-intensity walking for 40 min per day, 3 days per week, and weekly exercise counselling (n=58) - Usual care (n=58) | 12 weeks | - 64.76 - 63.57 | - 55.2 - 51.7 | HADS | HADS |
| Cugusi (2015)^39^ | Italy | Patients with Parkinson’s disease | 20 | - Nordic walking group twice per week for 1 hour each session (n=10) - Conventional care (n=10) | 12 weeks | - 68.1 - 66.6 | - 20 - 20 | BDI-II |  |
| Ferreira (2015)^40^ | Brazil | Community-dwelling elderly | 102 | - Supervised walking three times a week, with duration of 40–50 minutes for each session (n=34) - Respiratory training three times a week, with duration of 40–50 minutes for each session (n=34) - Social interaction control group three times a week (n=34) | 6 months | - 66.2 - 65.9 - 69.2 | - 68.18 - 87.5 - 86.3 | GDS | STAI (State) |
| Harris (2015)^41^ | UK | Patients aged 60–74 years registered at three general practices | 298 | - Primary care nurse physical activity consultations incorporating behaviour change techniques, pedometer step-count and accelerometer PA intensity feedback, and an individual PA diary and plan. (n=150) - Usual care (n=148) | 3 months |  | - 54 - 53.4 | GDS-15 | FEAR-4 |
| Hartescu (2015)^42^ | UK | Inactive people with insomnia | 41 | - Moderate-intensity physical activity brisk walking for at least 30 min per day, on at least 5 days of the week (n=20) - Wait-list control (n=21) | 6 months | - 59.5 - 60.1 | - 75 - 71.4 | BDI | STAI (Trait) |
| Abd El-Kader (2016)^43^ | Saudi Arabia | Subjects with Alzheimer’s disease | 59 | - Treadmill aerobic exercise (n=29) - No intervention (n=30) | 2 months | - 68.94 - 69.13 | - 30 - 25 | BDI |  |
| Abdelhamid (2016)^44^ | Egypt | Elderly subjects with mild depression | 100 | - A specialized program of date consumption in form of eating three dates daily (n=50) - Aerobic exercise in form of walking on a computerized treadmill for about twenty minutes with 5-10 min warming up and 5-10 min cooling down three times per week (n=50) | 24 weeks | - 63.38 - 62.84 | - 50 - 50 | GDS |  |
| Gokal (2016)^45^ | UK | Breast cancer patients undergoing chemotherapy | 50 | - Self-managed home-based moderate intensity walking intervention (n=25) - Usual care (n=25) | 12 weeks | - 52.08 - 52.36 |  | HADS | HADS |
| Picelli (2016)^46^ | Italy | Patients with Parkinson’s disease | 17 | - Treadmill training with 45-minute sessions 3 days/week (n=9) - No physical treatment (n=8) | 1 month | - 71.2 - 71.6 | - 44.4 - 50 | BDI |  |
| Shahabi (2016)^47^ | USA | Patients with irritable bowel syndrome | 35 | - Yoga 60 min biweekly (n=22) - Walking 60 min biweekly (n=13) | 8 weeks | - 34.7 - 39 | - 88.2 - 90 |  | STAI (State) |
| Tsianakas (2017)^48^ | UK | People with recurrent or metastatic cancer | 42 | - Recommendation to walk for at least 30 min on alternate days and attend a volunteer-led group walk weekly (n=21) - Standard care (n=21) | 12 weeks | - Men 65, women 60 - Men 66.2, women 58 | - 48 - 52 | DASS | DASS |
| Vancini (2017)^49^ | Brazil | Overweight/obese adults | 72 | - No intervention - 60-minute walking sessions three times per week - 60-minute Pilates sessions three times per week | 8 weeks | - 41.7 - 42.4 - 55.9 | - 60 - 95.24 - 95.45 | BDI | STAI |
| Van Schaardenburgh (2017)^50^ | Norway | Patients with intermittent claudication | 29 | - Calf raise exercise three times a day (n=14) - Traditional walking exercise group at least 30 minutes three times a week (n=15) | 8 weeks | - 66 - 70 | - 50 - 46.2 |  | Disease anxiety, CLAU-S |
| Bergman (2018)^51^ | Sweden | Overweight or obese healthy office workers | 80 | - Portable treadmill workstation with instruction to use the treadmill at a self-chosen walking speed (not running) for at least 1 h per day (n=40) - Control group continued to work as usual at their office desk (n=40) | 13 months | - 52.4 - 50.3 | - 55 - 55 | HADS | HADS |
| Coelho (2018)^52^ | Brazil | Adults with moderate to severe asthma | 37 | - Pedometer received the same exacerbation diary plus a pedometer (n=20) - Control group received a diary to register information about asthma exacerbations (n=17) | 12 weeks | - 45 - 47 | - 90 - 82.4 | HADS | HADS |
| Harris (2018)^53^ | UK | 45- to 75-year-old inactive adults | 1023 | - The nurse-supported pedometer-based walking programme (n=346) - The pedometer-based walking programme (n=339) - Usual activities (n=338) | 3 months |  | - 63.0 - 63.4 - 66.0 | HADS | HADS |
| Katz (2018)^54^ | USA | Patients under rheumatoid arthritis treatment | 96 | - Educational brochure and booklet only (n=28) - Educational booklet plus a pedometer and a diary to record daily step counts from the pedometer (n=34) - Educational booklet, pedometer and step diary, and individualized daily step targets. (n=34) | 21 weeks | - 59.1 - 55.9 - 50.2 | - 85.7 - 88.2 - 88.2 | PHQ-8 |  |
| Kuo (2018)^55^ | China (Taiwan) | Dwelling elderly 65 years and older | 41 | - Prescribed stepper walking program 30-minute twice per week (n=19) - Control group keeping routine activity and record activity in a diary (n=22) | 8 weeks | - 68.93 - 70.38 | - 80 - 85.7 | GDS-15 |  |
| Putra (2018)^56^ | Indonesia | Female adolescents aged 15-17 years | 64 | - Walking exercise daily for 1.6 km under 23 min, at a speed of 3.8 km/h on a treadmill 3 times/week (n=16) - Banana intake of 2 servings daily (130 g/serving) (n=16) - Walking exercise and banana intake (n=16) - Control not prescribed banana or walking exercise (n=16) | 2 weeks |  |  | BDI-II |  |
| Teng (2018)^57^ | China (Taiwan) | Heart failure patients | 90 | - Walking with breathing program twice daily (n=45) - Usual care (n=45) | 12 weeks | - 51.64 - 51.91 | - 15.6 - 24.4 | HADS | HADS |
| Abdelbasset (2019)^58^ |  | Patients with congestive heart failure-related depression | 46 | - Moderate-intensity continuous aerobic exercise (treadmill walking) 3 times per week for 40 to 50minutes (n=23) - Control group received only patient recommendations given by psychosocial unit without any physical exercise (n=23) | 12 weeks | - 53.4 - 52.9 | - 21.7 - 26.1 | PHQ-9 |  |
| Lin (2019)^59^ | China (Taiwan) | Patients with chronic obstructive pulmonary disease | 82 | - Breathing-based walking intervention about 30 min per day, 5 days a week (n=42) - Daily activity as usual with monthly telephone support (n=40) | 2 months | - 70.92 - 73.5 | Overall 4.8 | HADS | HADS |
| Miyamoto (2019)^60^ | Brazil | Women with primary Sjögren’s syndrome | 45 | - Supervise walking three times a week (n=23) - No intervention (n=22) | 16 weeks | - 53.4 - 51.3 | - 100 - 100 | BDI |  |
| Purnomo (2019)^61^ | Indonesia | Subject with central obesity | 80 | - Control group (n=20) - Brisk walking, thrice a week, increased from 20 to 40 minutes (n=20) - Relaxation, thrice a week, increased from 10 to 20 minutes (n=20) - Combination of brisk walking and relaxation, thrice a week (n=20) | 6 weeks | - 47.75 - 48.05 - 47.2 - 47.65 | - 20 - 25 - 20 - 20 |  | TMAS |
| Shi (2019)^62^ | USA | Adults with inadequate physical activity | 38 | - One-hour-per-week mindful walking intervention (n=17) - Control group received instructions to increase physical activity (n=21) | 4 weeks | - 52.7 - 46.5 | - 82.4 - 90.5 | 6-item Brief EDS |  |
| Suh (2019)^63^ | South Korea | People with chronic low back pain | 60 | - Flexibility exercise for 30∼60 minutes, 5 times a week (n=15) - Walking exercise for 30∼60 minutes, 5 times a week (n=15) - Stabilization exercise for 30∼60 minutes, 5 times a week (n=15) - Stabilization with walking exercise for 30∼60 minutes each, 5 times a week (n=15) | 6 weeks | - 53.54 - 54.15 - 57.4 - 54.75 | - 61.5 - 84.6 - 60 - 66.7 | BDI |  |
| Dougherty (2020)^64^ | USA | Participants with single- or dual-chamber implantable cardioverter defibrillator | 160 | - 8 week of home walking 1 hr/day 5 day/week, followed by 16 week of maintenance home walking for 150 min/week (n=84) - Usual care monitoring for health concerns and amount of exercise using monthly phone contact (n=76) | 24 weeks | - Adherent (n=48): 59.9 Nonadherent (n=36): 50.4 - 53.6 | - 20.2 - 25 | PHQ-9 | STAI |
| Rezola-Pardo (2020)^65^ | Spain | Older adults living in long-term nursing homes | 141 | - Individualized multicomponent group with strength and balance exercises twice a week lasting approximately an hour per session (n=41) - Individualized walking group up to 20 min per day (n=40) | 3 months | - 84.7 - 83.8 | - 63.4 - 67.5 | ADGS | ADGS |
| Sheshadri (2020)^66^ | USA | Dialysis patients | 60 | - Pedometers with weekly step goals (n=30) - Usual care (n=30) | 3 months | - Median 60 - Median 56 | - 37 - 7 | CES-D |  |
| Yentür (2020)^67^ | Turkey | Rheumatoid arthritis patients | 33 | - Pilates exercises were applied for 3 times a week with 45 min per session (n=11) - Walking on treadmill was applied for three times in a week with 30 min per session (n=11) - Combined Pilates and walking exercises (n=11) | 8 weeks | - 48.2 - 50.7 - 51.9 |  | BDI |  |
| Bade (2021)^68^ | USA | Patients with advanced stage lung cancer | 40 | - An accelerometer and individualized walking goals based on average daily step count during week 1 (n=20) - Usual care (n=20) | 12 weeks | - 66.55 - 63.2 | - 60 - 90 | PHQ-9 |  |
| Gjellesvik (2021)^69^ | Norway | Adult stroke survivors | 70 | - Standard care in combination with treadmill high-intensity interval training performed 3 times per week (n=36) - Standard care only with information about the benefits of high levels of physical activity (n=34) | 8 weeks | - 57.6 - 58.7 | - 41.7 - 41.2 | HADS | HADS |
| Hammer (2021)^70^ | USA | Patients without diabetes undergoing chemotherapy | 42 | - A prescribed walking program intervention group with three times per week, 30-min walking distance (n=21) - Control group with an exercise information handout (n=21) | 6 months | - 50.2 - 47.17 | - 93.3 - 100 | CES-D |  |
| Saavedra (2021)^71^ | Iceland | Healthy office worker volunteers | 47 | - Circuit training program of 3 sessions/week, with a duration of 30 min in the middle of the workday (n=18) - Brisk walk program of 3 sessions/week, with a duration of 30 min in the middle of the workday (n=18) - No intervention (not randomized) (n=11) | 12 weeks | - 44.8 - 45.6 - 43.2 | - 89 - 83 - 55 | DASS | DASS |
| Burgess (2022)^72^ | USA | Black patients with moderate to severe chronic back, hip, or knee pain | 380 | - Six telephone coaching sessions over 8–14 weeks, proactively delivered, using action planning and motivational interviewing to increase walking (n=193) - Usual care with a brochure (n=187) | 8-14 weeks/ 6 months | - 59 - 58 | - 25.4 - 30.5 | PHQ-8 | GAD-7 |
| Khalili (2022)^73^ | Iran | young-old women aged 60–74 years old | 70 | - Group walking program with 30 min/session, 3 sessions a week (n=35) - No intervention (n=35) | 8 weeks | - 64.2 - 64.2 | - 100 - 100 |  | SPAS |
| Noushad (2022)^74^ | Pakistan | Healthcare providers with post-traumatic stress | 262 | - Walking in nature at a moderate pace in 60 min sessions 5 times per week (n=131) - Nature-based sitting with 60 min five times per week (n=131) | 3 months | - 33.14 - 32.41 | - 55.7 - 45.8 | TSC | TSC&STAI |
| Reed (2022)^75^ | Canada | Coronary artery disease patients who underwent coronary revascularization procedures | 130 | - High-intensity interval training session with 45 min in duration (n=43) - Nordic walking session with 60 min in duration (n=44) - Moderate-to-vigorous intensity continuous training session with 60 min in duration (n=43) | 12 weeks | - 61 - 61 - 60 | - 16.3 - 16.3 - 13.6 | BDI-II |  |

GHQ=General Health Questionnaire; ADGS=Anxiety and Depression Goldberg Scale; AIMS=Arthritis Impact Measurement Scales; BAI=Beck Anxiety Inventory; BDI=Beck Depression Inventory; BDI-II=Beck Depression Inventory-II; BRMS=Bech-Rafaelsen Melancholy Scale; CES-D=Center for Epidemiologic Studies–Depression scale; CLAU-S=Claudication Scale; DASS=Depression Anxiety Stress Scales; EPDS=Edinburgh Postnatal Depression Scale; EDS= Edinburgh Depression Scale; FEAR=Frequency of anxiety, enduring nature of anxiety, alcohol or sedative use, restlessness or fidgeting; GAD=Generalized Anxiety Disorder; GDS=Geriatric Depression Scale; GWBS=General Well-Being Schedule; HADS=Hospital Anxiety and Depression Scale; HAM-D=Hamilton Rating Scale for Depression; PHQ=Patient Health Questionnaire; PMOS=Profile of Mood States; POMS-BI=Profile of Mood States, Bipolar Form; SPAS=Social Physique Anxiety Scale; STAI=State-Trait Anxiety Index; TMAS=Taylor Manifest Anxiety Scale; TSC=Trauma Symptom Checklist.

**Table S4.** Risk of bias of included trials evaluated using the Cochrane Risk of Bias tool

| Study | Random sequence generation (selection bias) | Allocation concealment (selection bias) | Blinding of outcome assessment (detection bias) | Incomplete outcome data (attrition bias) | Selective reporting (reporting bias) | Other bias | Overall bias |
| --- | --- | --- | --- | --- | --- | --- | --- |
| Minor (1989)^1^ | Unclear | Unclear | Unclear | Low | Low | Low | Unclear |
| Sexton (1989)^2^ | Unclear | Unclear | Unclear | Low | Low | Low | Unclear |
| Sinatra (1990)^3^ | Unclear | Unclear | Unclear | Low | Low | Low | Unclear |
| Cramer (1991)^4^ | Unclear | Unclear | Unclear | Low | Low | Low | Unclear |
| McNeil (1991)^5^ | Unclear | Unclear | Unclear | Unclear | Low | Low | Unclear |
| Palmer (1995)^6^ | Unclear | Unclear | Unclear | Low | Low | Low | Unclear |
| Stanton (1996)^7^ | Unclear | Unclear | Unclear | Unclear | Low | Low | Unclear |
| Moreau (1999)^8^ | High | High | Unclear | High | Low | Low | High |
| Nieman (2000)^9^ | Unclear | Unclear | Unclear | Low | Low | Low | Unclear |
| King (2002)^10^ | Low | Unclear | Unclear | Low | Low | Low | Unclear |
| Penninx (2002)^11^ | Unclear | Unclear | Low | High | Low | Low | High |
| Armstrong (2004)^12^ | Low | Low | Unclear | Low | Low | Low | Unclear |
| Motl (2005)^13^ | Low | Unclear | Unclear | Low | Low | Low | Unclear |
| Gary (2006)^14^ | Unclear | Unclear | Unclear | Low | Low | Low | Unclear |
| Knubben (2007)^15^ | Low | Low | Unclear | Low | Low | Low | Unclear |
| Bircan (2008)^16^ | Unclear | Unclear | Low | Low | Low | Low | Unclear |
| Chang (2008)^17^ | Unclear | Unclear | Unclear | Low | Low | Low | Unclear |
| Payne (2008)^18^ | Unclear | Unclear | Unclear | Unclear | Low | Low | Unclear |
| Smith (2008)^19^ | Low | Unclear | High | Low | Low | Low | High |
| Robichaud (2009)^20^ | Low | Unclear | Unclear | Low | Low | Low | Unclear |
| Breyer (2010)^21^ | Low | Unclear | Unclear | Low | Low | Low | Unclear |
| Chao (2010)^22^ | Low | Unclear | Unclear | Low | Low | Low | Unclear |
| Gary (2010)^23^ | Unclear | Unclear | Low | Low | Low | Low | Unclear |
| Collins (2011)^24^ | Low | Unclear | Low | Low | Low | Low | Unclear |
| McCaffrey (2011)^25^ | Unclear | Low | Low | Low | Low | Low | Unclear |
| Roshan (2011)^26^ | Unclear | Unclear | Unclear | Unclear | Low | Low | Unclear |
| Maki (2012)^27^ | Unclear | Unclear | Low | Low | Low | Low | Unclear |
| Ridsdale (2012)^28^ | Low | Low | Low | High | Low | Low | High |
| Ahmadi (2013)^29^ | Unclear | Unclear | Unclear | Low | Low | Low | Unclear |
| Faulkner (2013)^30^ | Unclear | Unclear | Unclear | High | High | Low | High |
| Jacobsen (2013)^31^ | Low | Unclear | Unclear | Low | Low | Low | Unclear |
| Pelssers (2013)^32^ | Unclear | Unclear | Unclear | Low | Low | Low | Unclear |
| Bernard (2014)^33^ | Low | Low | Unclear | Low | Low | Low | Unclear |
| Prakhinkit (2014)^34^ | Unclear | Unclear | Unclear | Low | Low | Low | Unclear |
| Van Hoecke (2014)^35^ | Unclear | Unclear | Unclear | Low | Low | Low | Unclear |
| Abedi (2015)^36^ | High | Unclear | Low | Low | Low | Low | High |
| Bellon (2015)^37^ | Low | Unclear | Low | High | Low | Low | High |
| Chen (2015)^38^ | Low | Low | Low | Low | Low | Low | Low |
| Cugusi (2015)^39^ | Unclear | Unclear | Unclear | Low | Low | Low | Unclear |
| Ferreira (2015)^40^ | Low | Unclear | Low | High | Low | Low | High |
| Harris (2015)^41^ | Low | Unclear | High | Low | Low | Low | High |
| Hartescu (2015)^42^ | Low | Unclear | Unclear | Low | Low | Low | Unclear |
| Abd El-Kader (2016)^43^ | Unclear | Unclear | Unclear | High | Low | Low | High |
| Abdelhamid (2016)^44^ | Unclear | Unclear | Unclear | Unclear | Low | Low | Unclear |
| Gokal (2016)^45^ | Low | Unclear | Unclear | Low | Low | Low | Unclear |
| Picelli (2016)^46^ | Low | Low | Low | Low | Low | Low | Low |
| Shahabi (2016)^47^ | Unclear | Unclear | Unclear | High | Low | Low | High |
| Tsianakas (2017)^48^ | Low | Unclear | Unclear | Low | Low | Low | Unclear |
| Vancini (2017)^49^ | Unclear | Unclear | Low | Low | Low | Low | Unclear |
| Van Schaardenburgh (2017)^50^ | Low | Unclear | Low | Low | Low | Low | Unclear |
| Bergman (2018)^51^ | Low | Unclear | Low | Low | Low | Low | Unclear |
| Coelho (2018)^52^ | Low | Unclear | Unclear | Low | Low | Low | Unclear |
| Harris (2018)^53^ | Low | High | High | Low | Low | Low | High |
| Katz (2018)^54^ | Low | Low | Unclear | Low | Low | Low | Unclear |
| Kuo (2018)^55^ | Low | Unclear | Low | Low | Low | Low | Unclear |
| Putra (2018)^56^ | Unclear | Unclear | Unclear | Low | Low | Low | Unclear |
| Teng (2018)^57^ | Low | Low | High | Low | Low | Low | High |
| Abdelbasset (2019)^58^ | Unclear | Unclear | Low | Low | Low | Low | Unclear |
| Lin (2019)^59^ | Low | Low | Low | Low | Low | Low | Low |
| Miyamoto (2019)^60^ | Unclear | Low | Low | Low | Low | Low | Unclear |
| Purnomo (2019)^61^ | Unclear | Unclear | Unclear | Low | Low | Low | Unclear |
| Shi (2019)^62^ | Low | Unclear | High | Low | Low | Low | High |
| Suh (2019)^63^ | Low | Low | Low | Low | Low | Low | Low |
| Dougherty (2020)^64^ | Unclear | Unclear | Unclear | High | Low | Low | High |
| Rezola-Pardo (2020)^65^ | Low | Low | Low | Low | Low | Low | Low |
| Sheshadri (2020)^66^ | Low | Low | Unclear | Low | Low | Low | Unclear |
| Yentür (2020)^67^ | Low | Unclear | Unclear | Low | Low | Low | Unclear |
| Bade (2021)^68^ | Unclear | Low | Unclear | Low | Low | Low | Unclear |
| Gjellesvik (2021)^69^ | Low | Unclear | Low | Low | Low | Low | Unclear |
| Hammer (2021)^70^ | Low | Unclear | Unclear | Low | Low | Low | Unclear |
| Saavedra (2021)^71^ | Unclear | Unclear | Unclear | Low | Low | Low | Unclear |
| Burgess (2022)^72^ | Low | Low | Low | High | Low | Low | High |
| Khalili (2022)^73^ | Low | Unclear | High | Low | Low | Low | High |
| Noushad (2022)^74^ | Low | Low | Unclear | Low | Low | Low | Unclear |
| Reed (2022)^75^ | Low | Low | Low | Low | Low | Low | Low |

**Table S5.** Category of different walking in the subgroup analysis when compared with inactive controls

| Study | Duration | Intensity | Increased intensity | Frequency | Minute | Pace | Format | Place | Following instruction | Prior training | Motivation | Pedo-  meter | Dropout rate | Study size | Mean  age | Baseline depressed |
| --- | --- | --- | --- | --- | --- | --- | --- | --- | --- | --- | --- | --- | --- | --- | --- | --- |
| Sinatra (1990)^3^ | 3-6m | NA | Yes | <5d/wk | NA | NA | NA | NA | No | No | No | No | >10 | <100 | 30-60 | No |
| Cramer (1991)^4^ | 3-6m | Moderate | No | >=5d/wk | 35-60 | Guided | Group | NA | Yes | No | No | No | >10 | <100 | 30-60 | No |
| McNeil (1991)^5^ | <3m | NA | Yes | <5d/wk | 35-60 | Guided | NA | Outdoor | No | No | No | No | 0-10 | <100 | >60 | Yes |
| Palmer (1995)^6^ | <3m | Moderate | Yes | NA | 10-30 | Guided | Group | Indoor | Yes | No | No | No | 0-10 | <100 | 30-60 | No |
| Stanton (1996)^7^ | 3-6m | Moderate | No | <5d/wk | 35-60 | Guided | Individual | NA | No | No | No | No | >10 | >=100 | 30-60 | No |
| Moreau (1999)^8^ | 3-6m | NA | Yes | <5d/wk | NA | Both | NA | NA | No | No | No | Yes | >10 | <100 | 30-60 | No |
| Chang (2008)^17^ | <3m | NA | No | >=5d/wk | 10-30 | Guided | NA | Indoor | Yes | No | No | No | 0-10 | <100 | 30-60 | No |
| Payne (2008)^18^ | NA | Moderate | No | <5d/wk | 10-30 | Guided | Individual | NA | No | No | No | Yes | 0-10 | <100 | >60 | No |
| Smith (2008)^19^ | <3m | NA | Yes | <5d/wk | 10-30 | Guided | NA | Indoor | No | Yes | Yes | No | NA | <100 | 30-60 | No |
| Robichaud (2009)^20^ | <3m | NA | No | <5d/wk | 10-30 | NA | NA | NA | Yes | No | Yes | No | 0-10 | <100 | 30-60 | No |
| Breyer (2010)^21^ | 3-6m | High | No | <5d/wk | 35-60 | Guided | NA | Outdoor | Yes | Yes | No | No | 0-10 | <100 | >60 | No |
| Chao (2010)^22^ | <3m | NA | No | NA | NA | Self-selected | Individual | NA | No | No | No | Yes | 0-10 | <100 | 30-60 | No |
| Gary (2010)^23^ | 3-6m | Moderate | Yes | <5d/wk | 35-60 | Guided | Individual | Outdoor | No | Yes | No | No | 0-10 | <100 | >60 | Yes |
| Collins (2011)^24^ | 3-6m | NA | Yes | <5d/wk | 35-60 | Guided | Both | NA | Yes | Yes | Yes | Yes | >10 | >=100 | >60 | No |
| Ahmadi (2013)^29^ | <3m | Mild-moderate | Yes | <5d/wk | 10-30 | Guided | Group | Indoor | Yes | No | No | No | 0-10 | <100 | 30-60 | No |
| Jacobsen (2013)^31^ | 3-6m | Moderate-high | No | <5d/wk | 10-30 | Guided | Individual | NA | Yes | Yes | No | Yes | >10 | >=100 | 30-60 | No |
| Pelssers (2013)^32^ | <3m | NA | Yes | <5d/wk | 10-30 | Guided | Group | NA | No | No | No | Yes | >10 | >=100 | >60 | No |
| Bernard (2014)^33^ | 3-6m | Moderate | Yes | <5d/wk | 35-60 | Guided | NA | Outdoor | Yes | No | No | No | >10 | >=100 | >60 | No |
| Prakhinkit (2014)^34^ | 3-6m | Mild-moderate | Yes | <5d/wk | 10-30 | Guided | NA | Indoor | Yes | No | No | No | 0-10 | <100 | >60 | Yes |
| Abedi (2015)^36^ | 3-6m | NA | Yes | NA | NA | Self-selected | Individual | NA | No | No | No | Yes | 0-10 | >=100 | 30-60 | Yes |
| Chen (2015)^38^ | 3-6m | Moderate | No | <5d/wk | 35-60 | Guided | Individual | NA | No | Yes | Yes | No | >10 | >=100 | >60 | No |
| Cugusi (2015)^39^ | 3-6m | Moderate-high | Yes | <5d/wk | 35-60 | Guided | Group | Outdoor | Yes | Yes | No | No | 0-10 | <100 | >60 | No |
| Harris (2015)^41^ | 3-6m | Moderate | Yes | NA | 10-30 | Self-selected | Individual | NA | No | Yes | Yes | Yes | 0-10 | >=100 | >60 | No |
| Hartescu (2015)^42^ | 3-6m | Moderate-high | No | >=5d/wk | 10-30 | Guided | Individual | NA | No | No | No | Yes | >10 | <100 | 30-60 | No |
| Abd El-Kader (2016)^43^ | <3m | Moderate | No | <5d/wk | 35-60 | Guided | NA | Indoor | Yes | No | No | No | >10 | <100 | >60 | No |
| Gokal (2016)^45^ | 3-6m | Moderate | Yes | >=5d/wk | 10-30 | Self-selected | Individual | NA | No | Yes | Yes | Yes | >10 | <100 | 30-60 | No |
| Picelli (2016)^46^ | <3m | NA | Yes | <5d/wk | 35-60 | Guided | NA | Indoor | Yes | No | No | No | 0-10 | <100 | >60 | No |
| Tsianakas (2017)^48^ | 3-6m | NA | No | <5d/wk | 10-30 | NA | Both | NA | No | No | Yes | No | >10 | <100 | >60 | No |
| Vancini (2017)^49^ | <3m | Moderate | No | <5d/wk | 35-60 | Guided | Individual | Outdoor | Yes | No | No | No | >10 | <100 | 30-60 | No |
| Bergman (2018)^51^ | 13m | NA | No | >=5d/wk | 35-60 | Self-selected | Individual | Indoor | No | No | Yes | No | >10 | <100 | 30-60 | No |
| Coelho (2018)^52^ | 3-6m | Moderate | Yes | >=5d/wk | 10-30 | Guided | Individual | NA | No | Yes | Yes | Yes | 0-10 | <100 | 30-60 | No |
| Harris (2018)^53^ | 3-6m | Moderate | Yes | >=5d/wk | 10-30 | Guided | Individual | NA | No | No | Yes/No | Yes | 0-10 | >=100 | NA | No |
| Kuo (2018)^55^ | <3m | Moderate | Yes | <5d/wk | 10-30 | Guided | Individual | Indoor | Yes | No | No | No | >10 | <100 | >60 | No |
| Putra (2018)^56^ | <3m | NA | No | <5d/wk | 10-30 | Guided | NA | Indoor | Yes | No | No | No | 0-10 | <100 | NA | No |
| Teng (2018)^57^ | 3-6m | NA | No | >=5d/wk | 10-30 | Guided | Group | Indoor | Yes | No | Yes | No | 0-10 | <100 | 30-60 | No |
| Abdelbasset (2019)^58^ | 3-6m | Moderate | No | <5d/wk | 35-60 | Guided | NA | Indoor | Yes | No | No | No | 0-10 | <100 | 30-60 | Yes |
| Lin (2019)^59^ | <3m | NA | No | >=5d/wk | 10-30 | NA | NA | NA | Yes | Yes | No | No | 0-10 | <100 | >60 | No |
| Miyamoto (2019)^60^ | 3-6m | High | Yes | <5d/wk | 35-60 | Guided | NA | Outdoor | Yes | No | No | No | >10 | <100 | 30-60 | No |
| Purnomo (2019)^61^ | <3m | Moderate | Yes | <5d/wk | 10-30 | Guided | NA | Outdoor | Yes | No | No | No | NA | <100 | 30-60 | No |
| Shi (2019)^62^ | <3m | Mild | No | <5d/wk | 35-60 | Guided | Both | Indoor | Yes | Yes | No | Yes | 0-10 | <100 | 30-60 | No |
| Dougherty (2020)^64^ | 3-6m | Moderate-high | Yes | >=5d/wk | 35-60 | Guided | Individual | NA | No | No | Yes | Yes | NA | >=100 | 30-60 | No |
| Sheshadri (2020)^66^ | 3-6m | NA | Yes | >=5d/wk | NA | Self-selected | Individual | NA | No | No | Yes | Yes | 0-10 | <100 | 30-60 | No |
| Bade (2021)^68^ | 3-6m | NA | Yes | NA | NA | Self-selected | Individual | NA | No | Yes | Yes | Yes | 0-10 | <100 | >60 | No |
| Gjellesvik (2021)^69^ | <3m | High | No | <5d/wk | NA | Guided | NA | Indoor | Yes | No | Yes | No | 0-10 | <100 | 30-60 | No |
| Hammer (2021)^70^ | 3-6m | High | No | <5d/wk | 10-30 | Guided | NA | NA | No | Yes | No | No | >10 | <100 | 30-60 | No |
| Burgess (2022)^72^ | NA | NA | No | NA | NA | NA | Individual | NA | No | Yes | Yes | Yes | >10 | >=100 | 30-60 | No |
| Khalili (2022)^73^ | <3m | NA | No | <5d/wk | 10-30 | NA | Group | Outdoor | Yes | No | No | No | >10 | <100 | >60 | No |
| Noushad (2022)^74^ | 3-6m | NA | No | >=5d/wk | 35-60 | Guided | NA | Outdoor | Yes | No | No | No | 0-10 | >=100 | 30-60 | No |

d/wk: days per week; HCP: Healthcare professional; HR: Heart rate; IAC: Inactive control; LEX: Light exercise; NA: Not available.

**Table S6.** Meta regression on the effect of walking on depressive and anxiety symptoms compared with inactive controls.

| **Form of walking** | **SMD (95% CI)** | ***P*** | **R^2^ (%)** |
| --- | --- | --- | --- |
| **Depressive symptoms (40 studies)** |  |  | 37.3 |
| Motivation (Yes vs. No) | 0.142 (-0.313, 0.596) | .54 |  |
| Pedometer (Yes vs. No) | 0.259 (-0.189, 0.706) | .26 |  |
| Dropout rate (>10% vs. 0-10%) | 0.134 (-0.288, 0.557) | .53 |  |
| Mean age (>60 vs. 30-60 years) | -0.304 (-0.717, 0.109) | .15 |  |
| Baseline depressive symptoms (Depressed vs. Non-depressed) | -1.158 (-1.851, -0.466) | .001 |  |
| **Anxiety symptoms (23 studies)** |  |  | 17.0 |
| Intervention duration (3-6 vs. <3 months) | 0.287 (-0.268, 0.842) | .31 |  |
| Following instructions during walking (Yes vs. No) | -0.098 (-0.628, 0.432) | .72 |  |
| Motivation (Yes vs. No) | 0.438 (-0.077, 0.952) | .10 |  |

SMD: standardized mean difference. Inactive control included no intervention, wait list, usual care, brief instructions on physical activity, and placebo meeting. A higher SMD means more depressive/anxiety symptoms.


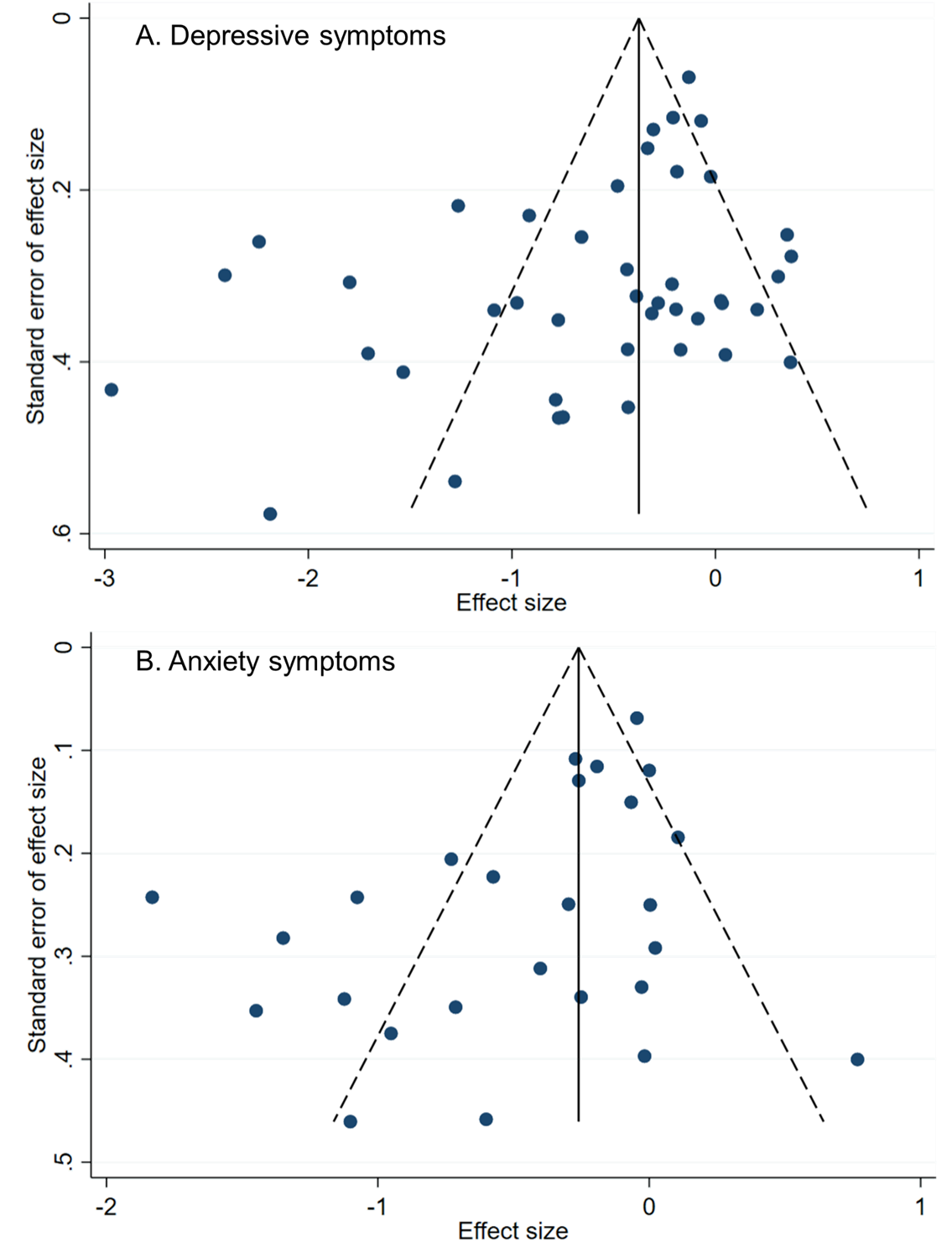


**Figure S1.** Funnel plots for the meta-analysis of the effect of walking on depressive and anxiety symptoms compared with inactive controls


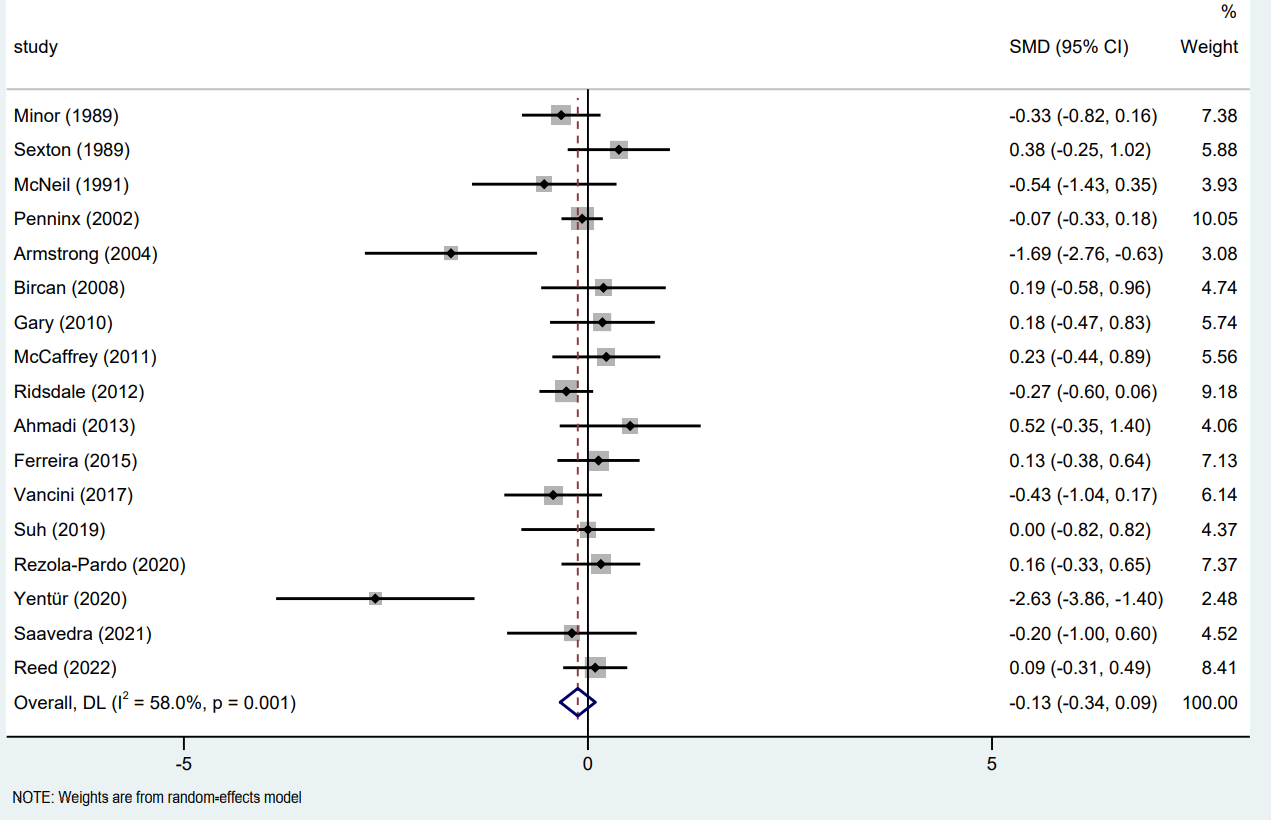


**Figure S2.** Forest plot for the effect of walking on depressive symptoms compared with active controls

SMD: standardized mean difference. Active control included other kind of moderate-intensity exercise (e.g., strengthening exercise, resistance exercise, swimming, cycling, jogging, Pilate, stabilization exercise), yoga, Tai Chi, meditation, cognitive behavior therapy, stress management training, art therapy, and social interaction. A higher SMD means more depression and anxiety symptoms.


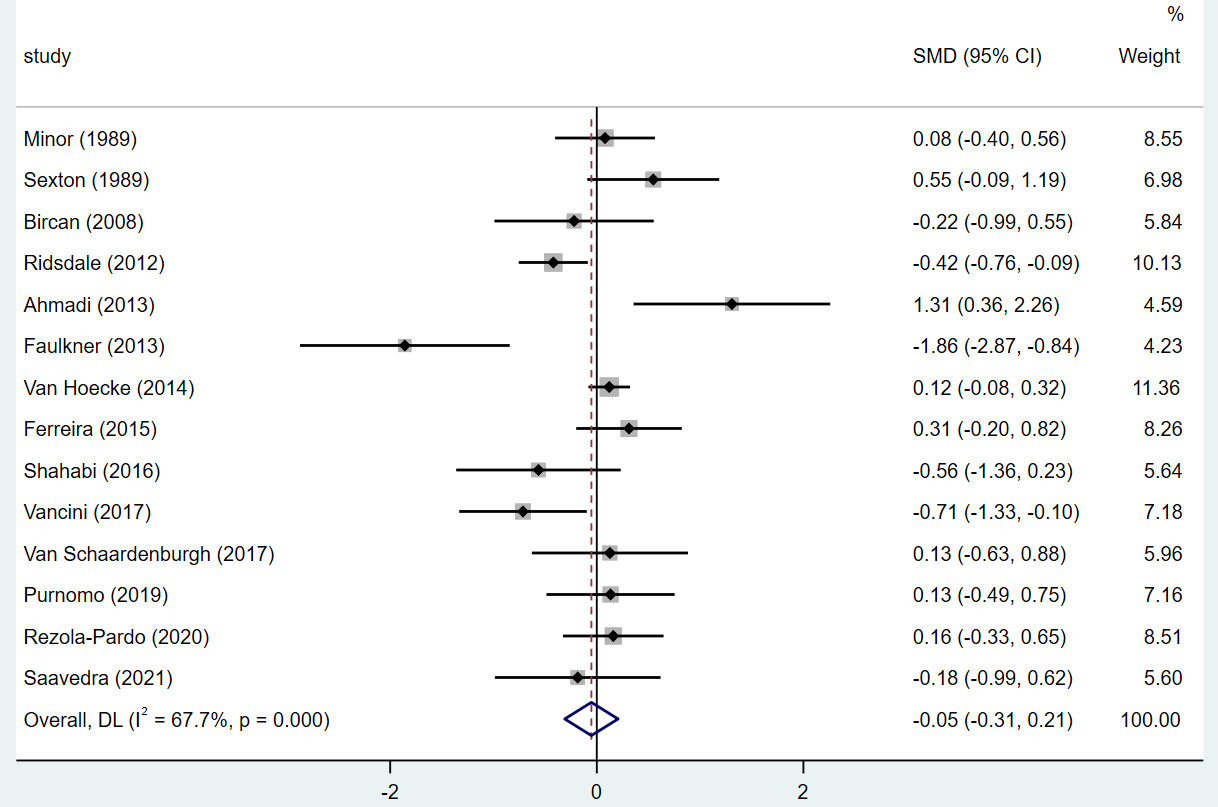


**Figure S3.** Forest plot for the effect of walking on anxiety symptoms compared with active controls

SMD: standardized mean difference. Active control included other kind of moderate-intensity exercise (e.g., strengthening exercise, resistance exercise, swimming, cycling, jogging, Pilate, stabilization exercise), yoga, Tai Chi, meditation, cognitive behavior therapy, stress management training, art therapy, and social interaction. A higher SMD means more depression and anxiety symptoms.


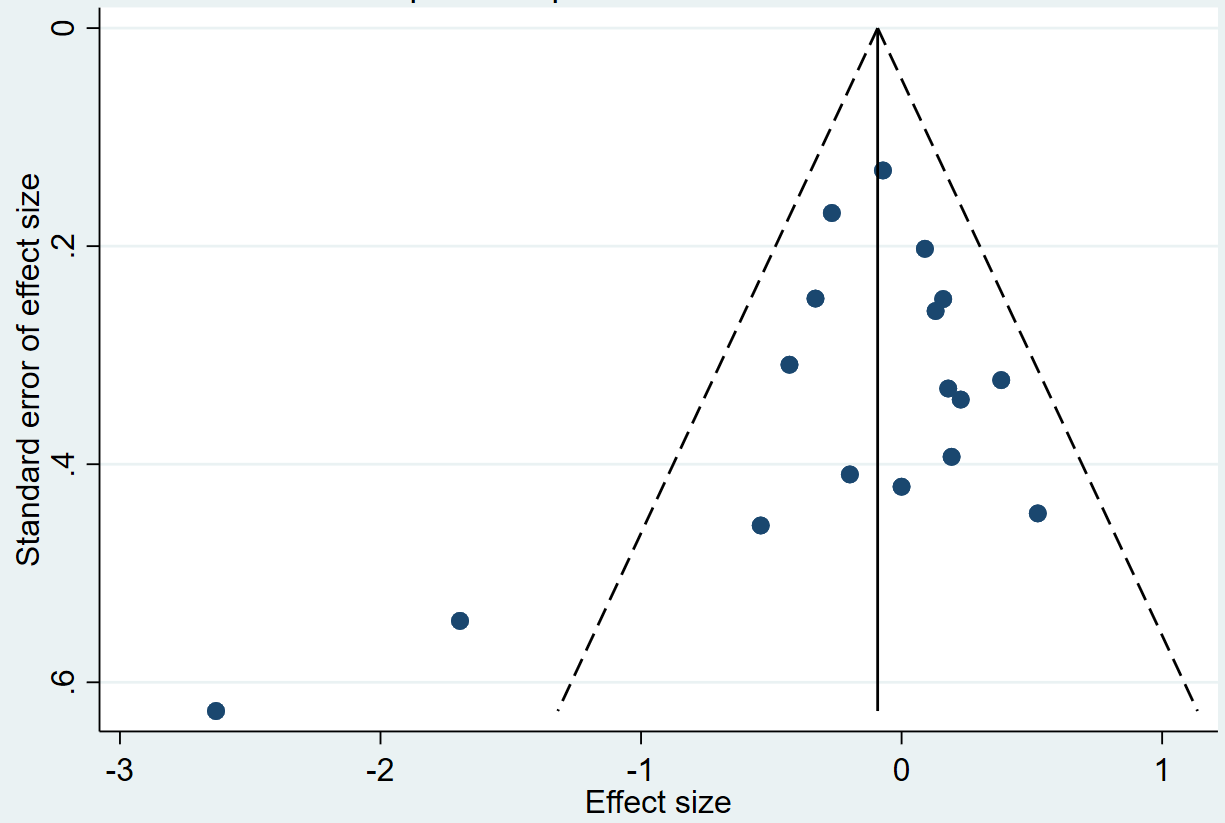

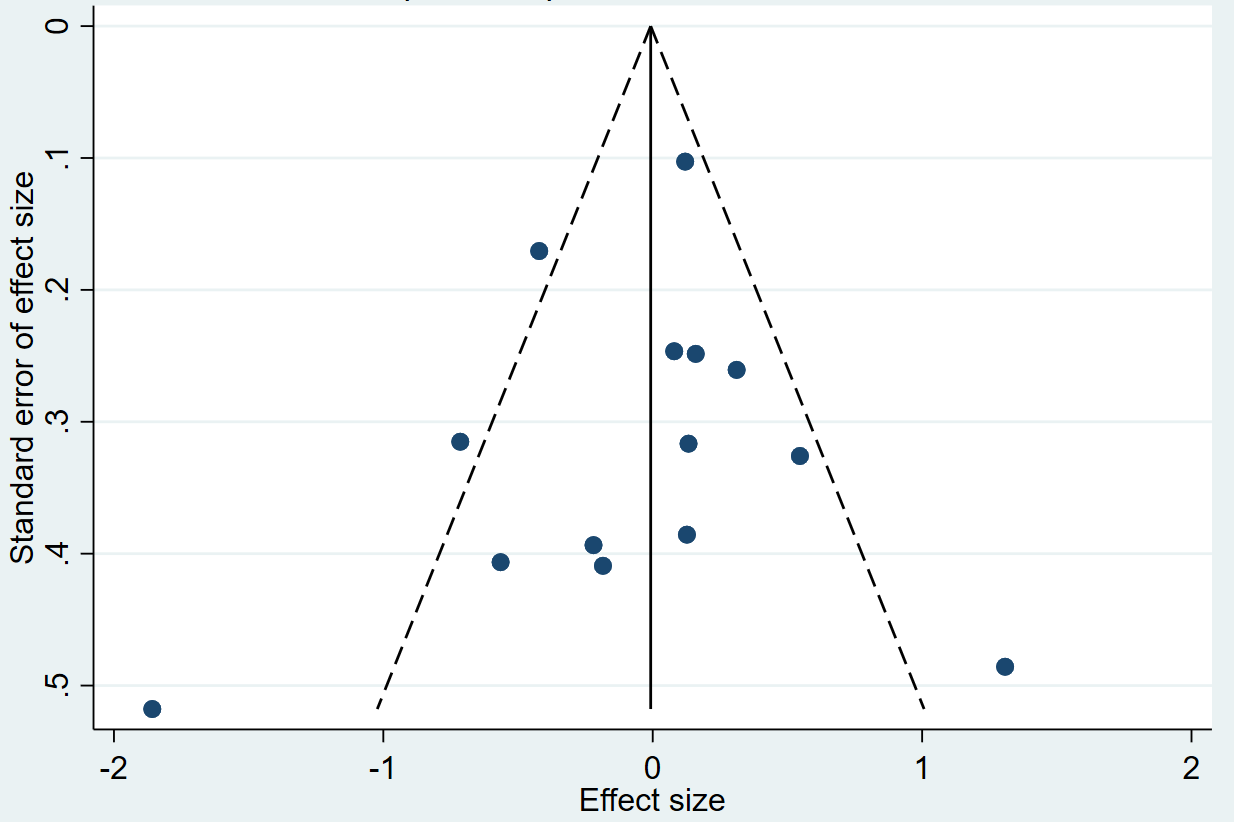


A. Depressive symptoms

B. Anxiety symptoms

**Figure S4.** Funnel plots for the meta-analysis of the effect of walking on depressive and anxiety symptoms compared with active controls


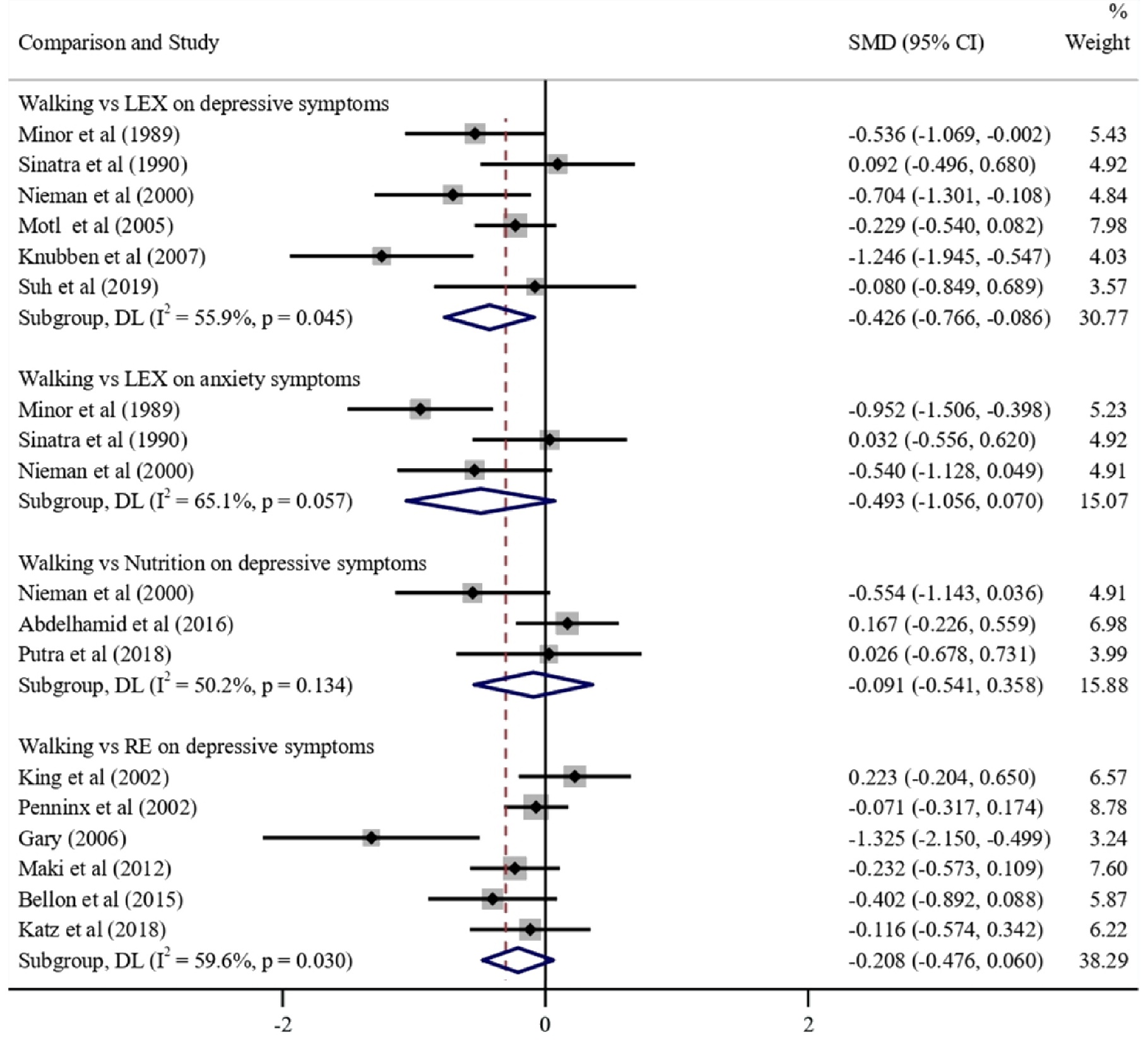


**Figure S5.** Forest plots for the effect of walking on depressive and anxiety symptoms compared with light exercise, nutrition or regular education

LEX: Light exercise; RE: Regular education; SMD: standardized mean difference.

**Reference**

1. Minor MA, Webel RR, Kay DR, Hewett JE, Anderson SK. Efficacy of physical conditioning exercise in patients with rheumatoid arthritis and osteoarthritis. *Arthritis & Rheumatism: Official Journal of the American College of Rheumatology* 1989; **32**(11): 1396-405.

2. Sexton H, Mære Å, Dahl N. Exercise intensity and reduction in neurotic symptoms: A controlled follow‐up study. *Acta Psychiatrica Scandinavica* 1989; **80**(3): 231-5.

3. Sinatra ST, Allen GJ, Camaione DN, Abraham A. Effects of Continuous Passive Motion, Walking, and a Placebo Intervention on Physical and Psychological Weil-Being. *Journal of Cardiopulmonary Rehabilitation and Prevention* 1990; **10**(8): 279-86.

4. Cramer SR, Nieman DC, Lee JW. The effects of moderate exercise training on psychological well-being and mood state in women. *Journal of psychosomatic research* 1991; **35**(4-5): 437-49.

5. McNeil JK, LeBlanc EM, Joyner M. The effect of exercise on depressive symptoms in the moderately depressed elderly. *Psychology and aging* 1991; **6**(3): 487.

6. Palmer LK. Effects of a walking program on attributional style, depression, and self-esteem in women. *Perceptual and Motor skills* 1995; **81**(3): 891-8.

7. Stanton JM, Arroll B. The effect of moderate exercise on mood in mildly hypertensive volunteers: a randomized controlled trial. *Journal of psychosomatic research* 1996; **40**(6): 637-42.

8. Moreau KL. The effects of walking volume on blood pressure in hypertensive postmenopausal women: The University of Tennessee; 1999.

9. Nieman DC, Custer WF, Butterworth DE, Utter AC, Henson DA. Psychological response to exercise training and/or energy restriction in obese women. *Journal of psychosomatic research* 2000; **48**(1): 23-9.

10. King AC, Baumann K, O'Sullivan P, Wilcox S, Castro C. Effects of moderate-intensity exercise on physiological, behavioral, and emotional responses to family caregiving: a randomized controlled trial. *The Journals of Gerontology Series A: Biological Sciences and Medical Sciences* 2002; **57**(1): M26-M36.

11. Penninx BW, Rejeski WJ, Pandya J, et al. Exercise and depressive symptoms: a comparison of aerobic and resistance exercise effects on emotional and physical function in older persons with high and low depressive symptomatology. *The Journals of Gerontology Series B: Psychological Sciences and Social Sciences* 2002; **57**(2): P124-P32.

12. Armstrong K, Edwards H. The effectiveness of a pram‐walking exercise programme in reducing depressive symptomatology for postnatal women. *International journal of nursing practice* 2004; **10**(4): 177-94.

13. Motl RW, Konopack JF, McAuley E, Elavsky S, Jerome GJ, Marquez DX. Depressive symptoms among older adults: long-term reduction after a physical activity intervention. *Journal of behavioral medicine* 2005; **28**(4): 385-94.

14. Gary R. Exercise self-efficacy in older women with diastolic heart failure: results of a walking program and education intervention. *Journal of gerontological nursing* 2006; **32**(7): 31-9; quiz 40.

15. Knubben K, Reischies FM, Adli M, Schlattmann P, Bauer M, Dimeo F. A randomised, controlled study on the effects of a short-term endurance training programme in patients with major depression. *British journal of sports medicine* 2007; **41**(1): 29-33.

16. Bircan Ç, Karasel SA, Akgün B, El Ö, Alper S. Effects of muscle strengthening versus aerobic exercise program in fibromyalgia. *Rheumatology international* 2008; **28**(6): 527-32.

17. Chang P-H, Lai Y-H, Shun S-C, et al. Effects of a walking intervention on fatigue-related experiences of hospitalized acute myelogenous leukemia patients undergoing chemotherapy: a randomized controlled trial. *Journal of pain and symptom management* 2008; **35**(5): 524-34.

18. Payne JK, Held J, Thorpe J, Shaw H. Effect of exercise on biomarkers, fatigue, sleep disturbances, and depressive symptoms in older women with breast cancer receiving hormonal therapy. Oncology nursing forum; 2008; 2008.

19. Smith PS, Thompson M. Treadmill training post stroke: are there any secondary benefits? A pilot study. *Clinical Rehabilitation* 2008; **22**(10-11): 997-1002.

20. Robichaud K. The effects of an exercise intervention on the psychological well-being of postpartum women. 2008.

21. Breyer M-K, Breyer-Kohansal R, Funk G-C, et al. Nordic walking improves daily physical activities in COPD: a randomised controlled trial. *Respiratory research* 2010; **11**(1): 1-9.

22. Chao PJ. A group randomized trial to examine the feasibility and effects of pedometer use and self-monitoring of daily walking in people with severe and persistent mental illnesses: [Honolulu]:[University of Hawaii at Manoa],[December 2010]; 2010.

23. Gary RA, Dunbar SB, Higgins MK, Musselman DL, Smith AL. Combined exercise and cognitive behavioral therapy improves outcomes in patients with heart failure. *Journal of psychosomatic research* 2010; **69**(2): 119-31.

24. Collins TC, Lunos S, Carlson T, et al. Effects of a home-based walking intervention on mobility and quality of life in people with diabetes and peripheral arterial disease: a randomized controlled trial. *Diabetes care* 2011; **34**(10): 2174-9.

25. McCaffrey R, Liehr P, Gregersen T, Nishioka R. Garden walking and art therapy for depression in older adults: a pilot study. *Research in gerontological nursing* 2011; **4**(4): 237-42.

26. Roshan VD, Pourasghar M, Mohammadian Z. The efficacy of intermittent walking in water on the rate of MHPG sulfate and the severity of depression. *Iranian Journal of Psychiatry and Behavioral Sciences* 2011; **5**(2): 26.

27. Maki Y, Ura C, Yamaguchi T, et al. Effects of intervention using a community‐based walking program for prevention of mental decline: a randomized controlled trial. *Journal of the American Geriatrics Society* 2012; **60**(3): 505-10.

28. Ridsdale L, Hurley M, King M, McCrone P, Donaldson N. The effect of counselling, graded exercise and usual care for people with chronic fatigue in primary care: a randomized trial. *Psychological medicine* 2012; **42**(10): 2217-24.

29. Ahmadi A, Arastoo AA, Nikbakht M, Zahednejad S, Rajabpour M. Comparison of the effect of 8 weeks aerobic and yoga training on ambulatory function, fatigue and mood status in MS patients. *Iranian Red Crescent Medical Journal* 2013; **15**(6): 449.

30. Faulkner J, Westrupp N, Rousseau J, Lark S. A randomized controlled trial to assess the effect of self-paced walking on task-specific anxiety in cardiac rehabilitation patients. *Journal of cardiopulmonary rehabilitation and prevention* 2013; **33**(5): 292-6.

31. Jacobsen PB, Phillips KM, Jim HS, et al. Effects of self‐directed stress management training and home‐based exercise on quality of life in cancer patients receiving chemotherapy: a randomized controlled trial. *Psycho‐Oncology* 2013; **22**(6): 1229-35.

32. Pelssers J, Delecluse C, Opdenacker J, Kennis E, Van Roie E, Boen F. “Every step counts!”: effects of a structured walking intervention in a community-based senior organization. *Journal of aging and physical activity* 2013; **21**(2): 167-85.

33. Bernard P, Ninot G, Bernard PL, et al. Effects of a six-month walking intervention on depression in inactive post-menopausal women: a randomized controlled trial. *Aging Ment Health* 2015; **19**(6): 485-92.

34. Prakhinkit S, Suppapitiporn S, Tanaka H, Suksom D. Effects of Buddhism walking meditation on depression, functional fitness, and endothelium-dependent vasodilation in depressed elderly. *The journal of alternative and complementary medicine* 2014; **20**(5): 411-6.

35. Van Hoecke A-S, Delecluse C, Bogaerts A, Boen F. Effects of need-supportive physical activity counseling on well-being: a 2-year follow-up among sedentary older adults. *Journal of Physical Activity and Health* 2014; **11**(8): 1492-502.

36. Abedi P, Nikkhah P, Najar S. Effect of pedometer-based walking on depression, anxiety and insomnia among postmenopausal women. *Climacteric* 2015; **18**(6): 841-5.

37. Bellon K, Kolakowsky-Hayner S, Wright J, et al. A home-based walking study to ameliorate perceived stress and depressive symptoms in people with a traumatic brain injury. *Brain injury* 2015; **29**(3): 313-9.

38. Chen H, Tsai C, Wu Y, Lin K, Lin C-C. Randomised controlled trial on the effectiveness of home-based walking exercise on anxiety, depression and cancer-related symptoms in patients with lung cancer. *British journal of cancer* 2015; **112**(3): 438-45.

39. Cugusi L, Solla P, Serpe R, et al. Effects of a Nordic Walking program on motor and non-motor symptoms, functional performance and body composition in patients with Parkinson’s disease. *NeuroRehabilitation* 2015; **37**(2): 245-54.

40. Ferreira L, Tanaka K, Santos-Galduróz RF, Galduroz JCF. Respiratory training as strategy to prevent cognitive decline in aging: a randomized controlled trial. *Clinical interventions in aging* 2015; **10**: 593.

41. Harris T, Kerry SM, Victor CR, et al. A primary care nurse-delivered walking intervention in older adults: PACE (pedometer accelerometer consultation evaluation)-Lift cluster randomised controlled trial. *PLoS medicine* 2015; **12**(2): e1001783.

42. Hartescu I, Morgan K, Stevinson CD. Increased physical activity improves sleep and mood outcomes in inactive people with insomnia: a randomized controlled trial. *Journal of sleep research* 2015; **24**(5): 526-34.

43. Abd El-Kader SM, Al-Jiffri OH. Aerobic exercise improves quality of life, psychological well-being and systemic inflammation in subjects with Alzheimer’s disease. *African health sciences* 2016; **16**(4): 1045-55.

44. Abdelhamid ZS, Serry ZM, Elnahas NM, Ammar NM. Serum serotonin response to aerobic exercise verus phoenix. *Int J PharmTech Res* 2016; **9**: 108-14.

45. Gokal K, Wallis D, Ahmed S, Boiangiu I, Kancherla K, Munir F. Effects of a self-managed home-based walking intervention on psychosocial health outcomes for breast cancer patients receiving chemotherapy: a randomised controlled trial. *Supportive Care in Cancer* 2016; **24**(3): 1139-66.

46. Picelli A, Varalta V, Melotti C, et al. Effects of treadmill training on cognitive and motor features of patients with mild to moderate Parkinson’s disease: a pilot, single-blind, randomized controlled trial. *Functional Neurology* 2016; **31**(1): 25.

47. Shahabi L, Naliboff BD, Shapiro D. Self-regulation evaluation of therapeutic yoga and walking for patients with irritable bowel syndrome: a pilot study. *Psychology, Health & Medicine* 2016; **21**(2): 176-88.

48. Tsianakas V, Harris J, Ream E, et al. CanWalk: a feasibility study with embedded randomised controlled trial pilot of a walking intervention for people with recurrent or metastatic cancer. *BMJ open* 2017; **7**(2): e013719.

49. Vancini RL, Rayes ABR, Lira CABd, Sarro KJ, Andrade MS. Pilates and aerobic training improve levels of depression, anxiety and quality of life in overweight and obese individuals. *Arquivos de neuro-psiquiatria* 2017; **75**: 850-7.

50. Van Schaardenburgh M, Wohlwend M, Rognmo Ø, Mattsson E. Calf raise exercise increases walking performance in patients with intermittent claudication. *Journal of vascular surgery* 2017; **65**(5): 1473-82.

51. Bergman F, Wahlström V, Stomby A, et al. Treadmill workstations in office workers who are overweight or obese: a randomised controlled trial. *The Lancet Public Health* 2018; **3**(11): e523-e35.

52. Coelho CM, Reboredo MM, Valle FM, et al. Effects of an unsupervised pedometer-based physical activity program on daily steps of adults with moderate to severe asthma: a randomized controlled trial. *Journal of Sports Sciences* 2018; **36**(10): 1186-93.

53. Harris T, Kerry S, Victor C, et al. A pedometer-based walking intervention in 45-to 75-year-olds, with and without practice nurse support: the PACE-UP three-arm cluster RCT. *Health Technol Assess* 2018; **22**(37): 1-274.

54. Katz P, Margaretten M, Gregorich S, Trupin L. Physical activity to reduce fatigue in rheumatoid arthritis: a randomized controlled trial. *Arthritis care & research* 2018; **70**(1): 1-10.

55. Kuo M-C, Chen C-M, Jeng C. A randomized controlled trial of the prescribed stepper walking program in preventing frailty among the dwelling elderly: application of comprehensive geriatric assessment. *Topics in Geriatric Rehabilitation* 2018; **34**(3): 223-333.

56. Putra ES, Wasita B, Anantanyu S. A randomised trial on walking exercise and banana consumption on self-reported depression symptoms among female adolescents in Surakarta, Indonesia. *Mal J Nutr* 2018; **24**(3): 467-73.

57. Teng H-C, Yeh M-L, Wang M-H. Walking with controlled breathing improves exercise tolerance, anxiety, and quality of life in heart failure patients: A randomized controlled trial. *European Journal of Cardiovascular Nursing* 2018; **17**(8): 717-27.

58. Abdelbasset WK, Alqahtani BA. A randomized controlled trial on the impact of moderate-intensity continuous aerobic exercise on the depression status of middle-aged patients with congestive heart failure. *Medicine* 2019; **98**(17).

59. Lin FL, Yeh ML, Lai YH, Lin KC, Yu CJ, Chang JS. Two‐month breathing‐based walking improves anxiety, depression, dyspnoea and quality of life in chronic obstructive pulmonary disease: A randomised controlled study. *Journal of clinical nursing* 2019; **28**(19-20): 3632-40.

60. Miyamoto ST, Valim V, Carletti L, et al. Supervised walking improves cardiorespiratory fitness, exercise tolerance, and fatigue in women with primary Sjögren’s syndrome: a randomized-controlled trial. *Rheumatology international* 2019; **39**(2): 227-38.

61. Purnomo KI, Doewes M, Suroto S, Murti B, Giri MKW. The combination effect of brisk walking and relaxation toward hs-crp and anxiety levels in subject with central obesity in Singaraja, Bali. *Bali Medical Journal* 2019; **8**(1): 294-8.

62. Shi L, Welsh RS, Lopes S, et al. A pilot study of mindful walking training on physical activity and health outcomes among adults with inadequate activity. *Complementary therapies in medicine* 2019; **44**: 116-22.

63. Suh JH, Kim H, Jung GP, Ko JY, Ryu JS. The effect of lumbar stabilization and walking exercises on chronic low back pain: A randomized controlled trial. *Medicine* 2019; **98**(26).

64. Dougherty CM, Burr RL, Kudenchuk PJ, Glenny RW. Aerobic exercise effects on quality of life and psychological distress after an implantable cardioverter defibrillator. *Journal of cardiopulmonary rehabilitation and prevention* 2020; **40**(2): 94.

65. Rezola-Pardo C, Rodriguez-Larrad A, Gomez-Diaz J, et al. Comparison between multicomponent exercise and walking interventions in long-term nursing homes: a randomized controlled trial. *The Gerontologist* 2020; **60**(7): 1364-73.

66. Sheshadri A, Kittiskulnam P, Lazar AA, Johansen KL. A walking intervention to increase weekly steps in dialysis patients: a pilot randomized controlled trial. *American Journal of Kidney Diseases* 2020; **75**(4): 488-96.

67. Yentür SB, Ataş N, Öztürk MA, Oskay D. Comparison of the effectiveness of pilates exercises, aerobic exercises, and pilates with aerobic exercises in patients with rheumatoid arthritis. *Irish Journal of Medical Science (1971-)* 2021; **190**(3): 1027-34.

68. Bade BC, Gan G, Li F, et al. Randomized trial of physical activity on quality of life and lung cancer biomarkers in patients with advanced stage lung cancer: a pilot study. *BMC cancer* 2021; **21**(1): 1-13.

69. Gjellesvik TI, Becker F, Tjønna AE, et al. Effects of high-intensity interval training after stroke (the HIIT stroke study) on physical and cognitive function: A multicenter randomized controlled trial. *Archives of Physical Medicine and Rehabilitation* 2021; **102**(9): 1683-91.

70. Hammer MJ, Eckardt P, Cartwright F, Miaskowski C. Prescribed walking for glycemic control and symptom management in patients without diabetes undergoing chemotherapy. *Nursing research* 2021; **70**(1): 6-14.

71. Saavedra JM, Kristjánsdóttir H, Gunnarsson SB, García-Hermoso A. Effects of 2 physical exercise programs (circuit training and brisk walk) carried out during working hours on multidimensional components of workers' health: a pilot study. *Int J Occup Med Environ Health* 2021; **34**(1): 39-51.

72. Burgess DJ, Hagel Campbell E, Hammett P, et al. Taking ACTION to Reduce Pain: a Randomized Clinical Trial of a Walking-Focused, Proactive Coaching Intervention for Black Patients with Chronic Musculoskeletal Pain. *Journal of general internal medicine* 2022: 1-9.

73. Khalili S, Shirinkam F, Ghadimi R, Karimi H. The effect of group walking program on social physique anxiety and the risk of eating disorders in aged women: A Randomized Clinical Trial study. *Applied Nursing Research* 2022; **64**: 151555.

74. Noushad S, Ansari B, Ahmed S. Effect of nature-based physical activity on post-traumatic growth among healthcare providers with post-traumatic stress. *Stress and Health* 2022; **n/a**(n/a).

75. Reed JL, Terada T, Cotie LM, et al. The effects of high-intensity interval training, Nordic walking and moderate-to-vigorous intensity continuous training on functional capacity, depression and quality of life in patients with coronary artery disease enrolled in cardiac rehabilitation: A randomized controlled trial (CRX study). *Progress in Cardiovascular Diseases* 2022; **70**: 73-83.
